# Unraveling gene regulatory networks from time-resolved gene expression data -- a measures comparison study

**DOI:** 10.1186/1471-2105-12-292

**Published:** 2011-07-19

**Authors:** Sabrina Hempel, Aneta Koseska, Zoran Nikoloski, Jürgen Kurths

**Affiliations:** 1 Interdisciplinary Center for Dynamics of Complex Systems, University of Potsdam, Campus Golm, Karl-Liebknecht-Str. 24, D-14476 Potsdam, Germany; 2 Potsdam Institute for Climate Impact Research (PIK), Telegraphenberg A 31, D-14473 Potsdam, Germany; 3 Department of Physics, Humboldt University of Berlin, Campus Adlershof, Newtonstr. 15, D-12489 Berlin, Germany; 4 Systems Biology and Mathematical Modeling Group, Max Planck Institute for Molecular Plant Physiology, Am Mühlenberg 1, D-14476 Potsdam, Germany; 5 Institute of Biochemistry and Biology, University of Potsdam, Karl-Liebknecht-Str. 25, D-14476 Potsdam, Germany; 6 Institute for Complex Systems and Mathematical Biology, University of Aberdeen, Aberdeen AB243UE, UK

## Abstract

**Background:**

Inferring regulatory interactions between genes from transcriptomics time-resolved data, yielding reverse engineered gene regulatory networks, is of paramount importance to systems biology and bioinformatics studies. Accurate methods to address this problem can ultimately provide a deeper insight into the complexity, behavior, and functions of the underlying biological systems. However, the large number of interacting genes coupled with short and often noisy time-resolved read-outs of the system renders the reverse engineering a challenging task. Therefore, the development and assessment of methods which are computationally efficient, robust against noise, applicable to short time series data, and preferably capable of reconstructing the directionality of the regulatory interactions remains a pressing research problem with valuable applications.

**Results:**

Here we perform the largest systematic analysis of a set of similarity measures and scoring schemes within the scope of the relevance network approach which are commonly used for gene regulatory network reconstruction from time series data. In addition, we define and analyze several novel measures and schemes which are particularly suitable for short transcriptomics time series. We also compare the considered 21 measures and 6 scoring schemes according to their ability to correctly reconstruct such networks from short time series data by calculating summary statistics based on the corresponding specificity and sensitivity. Our results demonstrate that rank and symbol based measures have the highest performance in inferring regulatory interactions. In addition, the proposed scoring scheme by asymmetric weighting has shown to be valuable in reducing the number of false positive interactions. On the other hand, Granger causality as well as information-theoretic measures, frequently used in inference of regulatory networks, show low performance on the short time series analyzed in this study.

**Conclusions:**

Our study is intended to serve as a guide for choosing a particular combination of similarity measures and scoring schemes suitable for reconstruction of gene regulatory networks from short time series data. We show that further improvement of algorithms for reverse engineering can be obtained if one considers measures that are rooted in the study of symbolic dynamics or ranks, in contrast to the application of common similarity measures which do not consider the temporal character of the employed data. Moreover, we establish that the asymmetric weighting scoring scheme together with symbol based measures (for low noise level) and rank based measures (for high noise level) are the most suitable choices.

## Background

Recent evidence from fully-sequenced genomes suggests that organismal complexity arises more from the elaborate regulation of gene expression than from the genome size itself [1]. It is not surprising that determining the interactions between genes, which gives rise to particular system's function and behavior, represents the grand challenge of systems biology [2]. In addition to structural information about the regulatory interactions, a comprehensive understanding of the dynamic behavior of these interactions requires specification of: (1) the type of regulation (*i.e.*, activation or inhibition) [3], (2) kinetics of interactions [4], and (3) the specificity of the interactions with respect to the investigated tissue and/or stress condition [5]. The elucidation of a complete network of regulatory interactions parameterized with kinetic information leading to a particular gene expression is, at present, still a challenging task even for well-studied model organisms whose networks have been partially assembled either for few selected processes and conditions or at the genome-wide level [6-9].

The ever-increasing throughput in experimental manipulation of gene activity coupled with the methods for quantitative assessment of transcriptome, proteome, and metabolome have begun to identify the effects of individual transcription factors, binding ligands, and post-translational modifications on regulated genes [10]. Moreover, such high-throughput transcriptomics data sets can be used to identify gene regulatory modules and entire networks. Understanding the complex network of gene regulatory interactions from a given transcriptome read-out necessitates the design, analysis, and testing of network-inference methods (so-called *reverse engineering *methods). These methods operate on two types of data sets from: (1) *static *perturbation experiments whose read-out is a pseudo steady-state expression level, and (2) *time-resolved *experiments yielding time series of gene expression.

Transcriptomics time series data hold the promise of identifying the dynamics of the key genes mapped into putative interactions and kinetic laws; consequently, the temporal information must not be neglected by the applied method for reverse engineering of gene regulatory networks. However, despite the decreasing costs of experiments relying on high-throughput technologies, systems biology studies still produce relatively short time series [11], largely due to the problems with gathering a big enough sample material and designing more complex experiments. In addition, time-resolved biological experiments usually involve sampling at irregular rates in order to capture processes spanning different time scales. These two challenges require a careful assessment of the existing methods for network inference from transcriptomics time series data. Moreover, most of the developed methods have been applied directly on real time series data without a prior assessment of their discerning capacity on a difficult synthetic benchmark [12].

The analysis of short time series is affected by the type of employed data representation. For instance, some approaches transform the discrete time series into continuous representations by different fitting methods; in addition, few studies have already considered transforming real valued time series into data-adaptive representations, including: symbols, strings, and trees (for a review, see [13]). Therefore, the extent to which a chosen data representation may affect the accuracy of the inferred networks should also be examined when assessing the strengths and weaknesses of different reverse engineering methods.

The simplest approach for network inference from time series data relies on applying similarity measures [12,14-27]. Methods borrowed from Bayesian inference [28-32], regression analysis [33], and econometrics models (*e.g.*, Granger causality [34-37]) have also been applied in this context. Although there are already two valuable reviews of methods for gene regulatory network (*GRN*) reconstruction from transcriptomics time series data [11,12], we believe that there is a need for a careful assessment of the existing reverse engineering methods based on similarity measures operating on short time series data.

**Figure 1:**
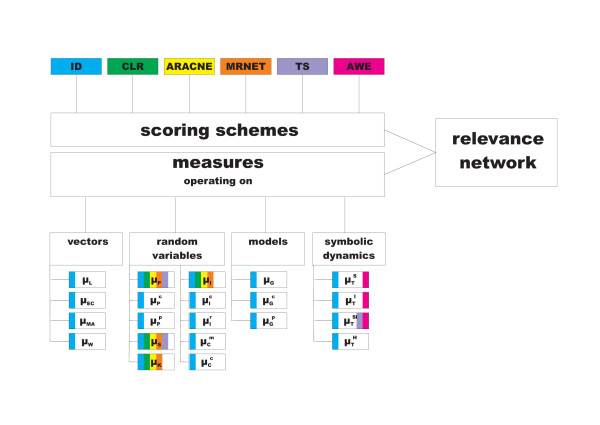
**Components of the relevance network algorithm for reverse engineering gene regulatory networks (*GRN*)**. The measures are grouped based on the representation on which they operate. Here, the different background colors indicate which combinations of scoring schemes and measures are studied. Altogether, there are 50 combinations included, because some measures can be further sub-divided.

For this purpose, we first divide the existing similarity measures (Table 1) into fourclasses based on the representation on which they operate, namely: vectors, random variables, models (*e.g.*, Granger causality), and symbols. We term the basic pairwise measures as simple, in comparison to their conditional and partial variants. The outcome of applying a similarity measure can further be refined via six scoring schemes: IDentity (*ID*), Algorithm for the Reconstruction of Accurate Cellular NEtworks (*ARACNE*), Context Likelihood of Relatedness (*CLR*), Maximum Relevance/minimum redundancy NETwork (*MRNET*), Time Shift (*TS*), and Asymmetric WEighting (*AWE*). The similarity measures and scoring schemes are schematically presented in Figure 1.

**Table 1:** Measures

MEASURE	"SIMPLE" (PAIRWISE)	CONDITIONAL	PARTIAL
Euclidean distance	*μ_EC_*, [63]	-	-
L*^s ^*Norm (here *s *= 10)	*μ_L_*, [64] (in literature *s *= 3)	-	-
Manhattan distance	*μ_MA_*, [64]	-	-
dynamic time warping distance	*μ_W_*, [43]	-	-
Pearson's correlation	*μ_P_*, [65]	, [22] (*)	, [23]
Spearman's correlation	*μ_S_*, [66]	-	-
Kendall's correlation	*μ _K_*, [67]	-	-
mutual information	*μ_I_*, [66]	, [25]	(new)
coarse-grained information rate	, [52] (*)	, [52] (*)	-
Granger causality index	*μ_G_*, [68]	, [34]	, [34]
symbol sequence similarity	, [38] (*)	-	-
mutual information of symbol sequence	, [38] (*)	-	-
mean of symbol sequence similarity and *^μ^I*	, [38] (*)	-	-
conditional entropy of symbol sequence	-	, [38] (*)	-

This table summarizes all measures of interaction included in this study. Those are marked by (*) which, to our knowledge, have not been applied to gene expression before.

We study the performance of the relevance network algorithm for *GRN *reconstruction, applied to synthetic gene expression data sets, and compare the capability of different combinations of 21 measures and 6 scoring schemes to detect/predict true and eliminate false links. A description of the data sets and the general definitions of the methods used in this study are given in detail in the Methods section.

Our contributions include: (1) an extensive systematic review and a comparison study which could serve as a basis for selecting a reverse engineering method based on a combination of a similarity measure and a scoring scheme suitable for a given expression data; in this context, we investigate not only the pairwise similarity measures, but also, where applicable, their respective conditional and partial variants; (2) introduction of approaches that are novel or borrowed from other fields, but have not yet been encountered in the field of network reconstruction; and (3) definition of a novel information-theoretic measure, the *residual mutual information*, and evaluation of its performance in unraveling gene regulatory interactions.

## Results

We investigate the performance of the relevance network algorithm applied to gene expression time series from a network of 100 genes in *E. coli *under optimal sampling conditions (noise-free, with an uniform sampling in time). Interpolation has not been applied to the time series at this point. We show and discuss the receiver operating characteristic (*ROC*) curves deduced for the basic algorithm with identity (*ID*) scoring scheme in combination with all measures included in our investigation, and for the five additional scoring schemes - *CLR*, *ARACNE*, *MRNET*, *TS *and *AWE *- with selected measures. Subsequently, since optimal sampling conditions are never achieved, the performance of the similarity measures and scoring schemes is additionally investigated on noisy data. The role of sampling and interpolation on the performance is discussed as well. Moreover, the influences of network properties (*e.g.*, size and degree) are shown using two additional networks, a yeast network composed of 100 genes from *S.cerevisiae *and a network of 200 genes from *E. coli*.

### Identity scoring scheme

The basic relevance network algorithm (scoring with unit matrix) is used to compare the performance of all measure's classes.

#### Measures operating on vectors

Additional file 1, Figure S1 shows the efficiency of the reconstruction of links based on classical distance measures and the dynamic time warping. In general, none of these measures is able to avoid false positives on a larger scale without loosing most of the true interactions. On the other hand, the *ROC *curves are rather flat for high false positive rates, which implies that these measures could be useful initially to determine connections which are not present in the network. All of the curves shown in Additional file 1, Figure S1 are smooth, meaning that the prediction of links is not very sensitive to the explicit choice of the threshold. From this analysis, we can discriminate that the *L^s ^*norm (with *s *= 10, equating the length of the time series) performs best in reconstructing the network. These results outperform the Euclidean (*L*^2 ^norm) and the Manhattan (*L*^1 ^norm) distance, which can be explained by the fact that the *L^s ^*weights large distances more heavily. The dynamic time warping fails for the investigated data, which is most likely a result of the coarse sampling and the complexity of the network.

#### Measures operating on random variables

Furthermore, the *ID *scoring scheme is evaluated using several measures which employ time series represented via random variables. In particular, we examine in detail the performance of correlation and information-theoretic measures.

**Figure 2:**
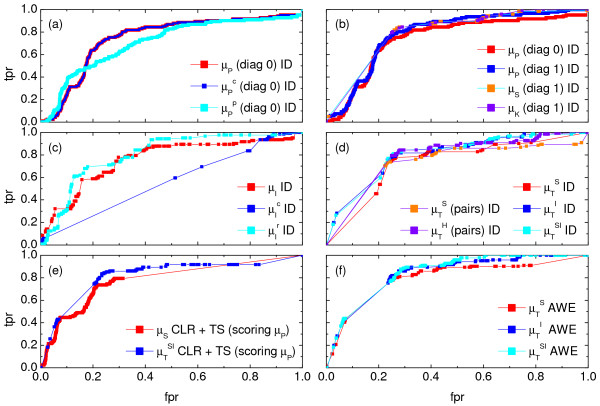
**Performance of various similarity measures (noise-free case)**. (a) *ROC *curves obtained for the *ID *scoring scheme using the simple, conditional and partial Pearson correlation (*μ_P_*, , ), where the diagonal of the cross-correlation matrix is set to 0. (b) *ROC *curves using the *ID *scoring scheme and different correlation coefficient, such as the simple Pearson correlation coefficient, where the diagonal of cross-correlation matrix is once 0 (*μ_P _*(*diag*0)), and another time the diagonal is 1 (*μ_P _*(*diag*1)). Furthermore, the *ROC *curves using the Spearman (*μ_S _*(*diag*1)) and the Kendall (*μ_K _*(*diag*1)) correlation coefficient, where the diagonal is 1 in both cases, are shown. (c) Evaluation of the *ID *scoring scheme using information-theoretic measures: simple, conditional and residual mutual information (*μ_I_*,  and ). (d) Evaluation of the *ID *scoring scheme using measures based on symbolic dynamics: symbol sequence similarity (), the mutual information of the symbol sequences () and the mean of these both (), as well as the symbol sequence similarity of pairs of time points ( (*pairs*)) and the conditional entropy of the symbols obtained from the pairs of time points ( (*pairs*)). (e) The corresponding *ROC *curves illustrating the performance of the Time Shift scoring scheme using the Pearson correlation *μ_P_*, applied in addition to the *CLR *(measure: *μ_S_*) and the *AWE *(measure: ) scoring scheme. (f) Performance of the *AWE *algorithm using the selected symbol based measures included in the this study, for example *ROC *curves for the symbol sequence similarity (), the mutual information of the symbol sequences (), and the mean of these both ().

In the case of the linear Pearson correlation (*PC*) coefficient, as shown in Figure 2(a), we obtain almost identical results from the simple and the conditional (*CPC*) measure, although the *CPC *is expected to eliminate indirect interactions. However, this does not mean that there are no indirect links wrongly deduced by the linear *PC*. The problem here is rooted in the estimation of the conditional probabilities, which is barely reliable for 10 time points.

Even if a basic significance test is included -- for example the data is reshuffled 100 times, then the measures for the randomized series are calculated, and the results are compared to those obtained from the original time series -- the results do not change significantly (Additional file 1, Figure S2). The partial Pearson correlation, on the other hand, shows better results for low false positive rates, but looses its accuracy when high true positive rates are reached. Additionally, the results obtained from the *PPC *are less significant (in terms of the reshuffled time series). Removing links which have no significant values of the correlation leads to an almost random prediction from the partial Pearson correlation.

As we cannot infer self-regulation by analyzing the similarity of expression series, the diagonal of the correlation matrix was set to zero in our computations above (by definition it is one). Comparing the reconstruction efficiency of the linear *PC *with that of the rank correlations (diagonal equals to one), we observe that the *ROC *curve shown in Figure 2(b) is smoother for the Pearson correlation than the curves obtained from the rank correlations. Hence Pearson's correlation measure is less sensitive to the choice of the threshold, whereas the rank correlations can achieve a slightly better overall performance.

Next, we investigate the efficiency of the *ID *scoring scheme considering information-theoretic measures. In general, we observe that the resulting reconstruction strongly depends on the method chosen for the estimation of entropies. Here we present the results obtained using the *R*-package "*infotheo*" (in particular the Miller-Madow asymptotic bias corrected empirical estimator) since, for short time series, it yields better estimates of the entropy than the *R*-package "*entropy*". Besides the basic pairwise mutual information (*MI*), we also investigated the conditional mutual information (*CMI*) and the residual mutual information (*RMI*) in order to reduce the number of false positive links. All these measures result in *ROC *curves which are more or less discontinuous. This is a finite size effect, as the time series are very short, and thus the estimation of the *MI *(entropies) becomes problematic.

We find a quite different behavior of the *ROC *curves, as shown in Figure 2(c), in specific regions of the *ROC *space. The simple mutual information results in a flat and comparatively smooth *ROC *curve for high false positive rates. This means that the measure allows removing about 60% of the false positives, by loosing approximately 10% of the true links. An even better performance in the same *ROC *space region can be achieved using the residual mutual information, which we proposed as a partial mutual information measure to distinguish indirect from direct (linear) relationships between triplets of genes. In contrast to this, the conditional *MI *results in a more discontinuous curve for high *fpr*: here, the ratio of the true and false positive rate is nearly the same as observed for a random prediction. In principle, the *CMI *is stricter in removing indirect links as it also can detect nonlinear interactions. However, the conditional probabilities cannot be estimated sufficiently well from 10 time points. Hence, the conditional *MI *fails for (the investigated) short data sets in the region of high false positive rates.

Additionally, when looking at the region of low *fpr*, we observe that the *ROC *curve of the simple *MI *becomes more discontinuous than for the high *fpr*. The true positive rate decreases significantly for slightly reduced threshold values, in the region around 30% and 15% of the false positives. This is manifested as jumps in the curve due to which this measure is rather sensitive to the choice of the threshold if low false positive rates are to be achieved. In contrast to this, the residual mutual information results in a smoother curve for low false positive rates than the simple *MI*, indicating that the measure is less sensitive to the choice of threshold, although the curve exhibits smaller jumps as well. In the region of *fpr *< 10% the performance of the *RMI *decreases slightly compared to the simple measure. The conditional mutual information on the other hand, achieves only very low false positive rates, which also lead to low true positive rates (up to about 5%). Tuning the threshold to allow for slightly higher values of the *fpr *the *ROC *curve of the *CMI *immediately jumps to 50% of false positives. Hence, the region between about 3% and 50% of false positive links is not achievable using the considered conditional measure.

We also implemented a basic significance test for the mutual information measures by reshuffling the time series 100 times, calculating the measure for the randomized series, and comparing the results to those obtained for the original time series. The associated *ROC *curves are shown in Additional file 1, Figure S3. With respect to the significance, the reconstruction efficiency of the simple and, in particular, the residual mutual information decreases, since the inferred degree of interaction for most of the gene pairs is not significant in the specified sense. In contrast to this, with the significance test, the quality of the prediction obtained from the *CMI *increases slightly, but its overall performance is still deficient.

That evaluation leads to the conclusion that (from the *MI *measures) only the simple and the residual mutual information can provide a sufficient reconstruction efficiency using the IDentity scoring scheme. This holds true only in the case that we do not rely on the simple significance test.

Investigating the performance of the coarse-grained measures on the short gene expression time series, we obtained *ROC *curves which look almost the same as expected for a complete random linking in the network, as illustrated in Additional file 1, Figure S4. Even though the coarse-grained measures are in principle promising for the inference of interdependency from time series of intermediate length, they are not applicable in our case. The reason for this is the limited number of available time points which makes not only the estimation of the *MI*, but also the identification of a proper time lag a very challenging task. Interpreting the *CCIR *as a distance, and not as a similarity measure, leads to an increase of the inferred true positives. However, the predictive power of the measure remains very low.

#### Model-based measures

The evaluation of the *ID *scoring scheme using model-based measures (Granger causality in this case) leads to an almost random prediction of links (the associated *ROC *curves are shown in Additional file 1, Figure S5). Hence, the Granger causality (*GC*) measure is not suitable for the reconstruction of *GRN*, when only very short gene expression time series are available. This is due to the fact that the results of the *GC *index depend strongly on the model estimation. An *AR *model has to be estimated for the given data set, whose order is determined based on the Akaike information criterion. However, this seems to be insufficient, since the *AIC *usually requires a higher order model (due to the high variability of the data), whereas the expression time series are in general very short.

#### Measures operating on symbolic dynamics

Next, we use the principle of order patterns to derive symbol sequences from the time series [38]. As already shown in general nonlinear time series analysis, the symbol based measures show a good overall performance in reverse engineering.

The *ROC *curves (Figure 2(d)) obtained for these measures are rather smooth and flat for false positive rates larger than 30%, which means that only a small portion of links is lost when reducing the false positive rates down to this value. Consequently, the results are robust to the choice of threshold in this particular region of the *ROC *space. However, the *ROC *curves become less smooth for lower values of the false positive rates. This implies that false positive rates smaller than 20% are barely possible to achieve. The best overall performance has been found here for the combination of symbol sequence similarity and mutual information of the symbol sequences (*SySimMI*), as well as for the mutual information of the symbol sequences (*SyMI*). The latter outperforms the simple *MI *of the time series themselves, as the length of the series used to estimate the measure is much longer in the case of the symbolic dynamics. Additionally, the conditional entropy of the symbol vectors obtained from pairs of time points shows results similar to the *SySimMI *and the *SyMI *in a wide range of the *ROC *space.

### Symmetric scoring schemes - *CLR*, *ARACNE *and *MRNET*

Next, we evaluate the possibility for reconstruction of the underlying *E. coli *(sub)network based on the three modifications of the relevance network algorithm (Algorithm 1, given in the Methods section) as implemented in the "*minet*"-package, namely the *CLR*, the *ARCANE *and the *MRNET*. All three algorithms represent extensions of the basic relevance network approach, to the effect that they introduce additional scoring rules for the pairwise weighting of the interactions in order to reduce the amount of links that are falsely detected.

In Additional file 1, Figure S6, we present the results, in terms of the *ROC *curves, which we obtain using the different scoring schemes and in all cases the default weights of the pairwise interactions, namely, the squared Spearman's correlation for every set of pairs. As the algorithms implemented in the "*minet*" are designed to reduce the number of false positives, high false positive rates (of more than about 50%) do not occur here, unless all interactions are set as links. Moreover, the *MRNET *and the *CLR *result in *ROC *curves which are not smooth, meaning that their capability to reconstruct particular links is limited and strongly dependent on a proper choice of the threshold *τ*. The *ARACNE*, on the other hand, is restricted to an almost fixed *fpr*-*tpr *value.

### Asymmetric scoring schemes - *TS *and *AWE*

As none of the previously described scoring schemes is able to indicate directionality from symmetric measures, we include in this study, and to our knowledge for the first time in *GRN *reconstruction, an evaluation of the performance of the Time Shift (*TS*) as a symmetry-breaking scoring scheme. We show the results of this modification of the relevance network algorithm removing the links which are falsely detected by the *CLR *(measure: *μ_ρ_*) or the *AWE *(measure: ). However, unraveling the directionality of interaction (between pairs of genes) using the correlation of the delayed time series has shown to decrease the maximal achievable true positive rates.

The slope of the *ROC *curves (shown in Figure 2(e)) indeed does not change much in comparison to the results of the *CLR *and the *AWE *scoring scheme. Moreover, if combined with the Pearson correlation of the delayed time series, the *ROC *curve obtained from the *CLR *becomes considerably smoother (compared to the curve shown in Additional file 1, Figure S6) and hence, the prediction is less sensitive to the choice of a threshold. The same does not hold true for the *ROC *curve obtained from the application of the *TS *scoring scheme in addition to the *AWE*. Instead, this curve becomes flatter and is slightly shifted towards lower false positive rates in comparison to the corresponding curve in Figure 2(f). This implies that, while for low *fpr *the curve looks basicly the same, in the intermediate range of the *ROC *space (*fpr *about 0.15 to 0.45) similar *tpr *values can be obtained for lower *fpr*. However, in the range of high *fpr*, the maximal achievable *tpr *value is lower. Hence, true positive rates of approximately 80% can be achieved with lower costs, as the according number of false positives is in general smaller when the *TS *scoring scheme is used. On the other hand, as already mentioned, the quality of the link detection becomes worse for higher false positive rates (more than about 40%) compared to the corresponding results of the *AWE *itself. The true positive rate in the *ROC *curve in Figure 2(e) is almost constant in this region of the *ROC *space.

Similar to the *TS*, the *AWE *scoring scheme aims at breaking symmetries and thus allows extraction of information about the directionality of interaction from symmetric measures. However, a detailed comparison of the reconstruction efficiency of the *AWE *using different symbolic dynamics measures shows that in contrast to the *TS *scoring scheme, *AWE *does not decrease the maximal achievable true positive rates. Instead, the *ROC *curves, as shown in Figure 2(f), become more flat for high false positive rates compared to the curves obtained for the basic algorithm with the *ID *scoring scheme using symbolic dynamics (Figure 2(d)). Hence true positive rates of more than 80% are achievable by the *AWE *algorithm with much lower costs than with the *ID *scoring scheme. On the other hand, the *ROC *curves obtained from *AWE *are more steep for low false positive rates. This implies that here true positive rates up to approximately 45% can be achieved with false positive rates of less than 10%. Furthermore, the curves shown in Figure 2(f) are much smoother in comparison to those in Figure 2(d), indicating that the reconstruction is less sensitive to the choice of a particular threshold.

### Influence of noise

In general, noise-free expression measurements cannot be achieved in real experiments: In fact, intermediate and high noise level are not rare. Thus, in order to account for stochasticity in the time series, and particularly to establish the robustness of the ranking of the investigated similarity measures, we additionally evaluate the *ROC *curves for noise intensity of 0.3.

**Figure 3:**
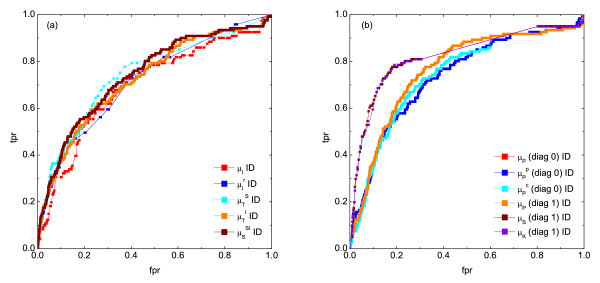
**Performance of various similarity measures for noisy data (noise level 0.3)**. The plot shows *ROC *curves of (a) mutual information (*μ_I_*), residual mutual information (), symbol sequence similarity (), mutual information of the symbol sequences () and the mean of these two (), and (b) Pearson correlation (*μ_P_*), partial Pearson correlation (), conditional Pearson correlation (), Spearman correlation (*μ_S_*) and Kendall correlation (*μ_K_*).

As expected, the measures which failed in the noise-free case (*e.g.*, *DTW*, *CMI*, the coarse-grained information rate, and the Granger causality measures) did not improve their performance, as shown in Additional file 1 Figure S7. On the other hand, the measures based on vectors yield very robust results with respect to noise (Additional file 1, Figure S7). However, since the performance of these measures was already insufficient in the noise-free case, its general overall ranking does not improve significantly. We additionally noticed that the measures which performed best in the case of optimal sampling conditions, such as *MI*, *RMI*, correlation and symbol based measures differ in their robustness against noise, as illustrated in Figure 3. For example, the reconstruction efficiency of the simple and the conditional Pearson's correlation slightly decreases, while that of partial Pearson's correlation slightly increases. Hence, all three measures result basically in the same *ROC *curves, meaning one can abandon the more computationally intensive calculation of partial and conditional Pearson's correlation under these circumstances. Furthermore, *MI *and *RMI *both lose their accuracy as noise increases, and the corresponding *ROC *curves resemble those of the Pearson's correlation. However, the relation between both measures stays the same (*RMI *performs slightly better than *MI*).

The reconstruction efficiency for the symbol based measures decreases significantly as well, which holds true in particular for the mutual information of symbol sequences (as noise affects the inference of a symbol sequence using order pattern, as well as the binning process for *MI *calculation). However, apart from that, the *ROC *curves obtained for the symbol based measures are more continuous for noisy data than those in the noise-free case, which implies that the reconstruction process in this case is less influenced by the choice of a particular threshold.

A similar behavior is observed for the rank correlation coefficients. However, the shape of the curves appears more robust under the influence of noise than it is the case for the symbol based measures. Hence, the rank based measures represent the most suitable similarity measures to study the interrelation among short time series at high noise levels.

Finally, we observe that the *CLR *and the *AWE *are the most robust scoring schemes with respect to noise, whereas *ARACNE *fails for short and noisy time series.

A detailed analysis on the reconstruction efficiency of the top-ranking measures and scoring schemes under various stochastic conditions is considered in the Discussion section. Additionally, the performance as a function of the length of the time series and the noise intensity can be found in the Additional files.

### The role of interpolation and sampling

Due to the fact that time-resolved gene expression data are usually quite coarsely sampled, general assumptions upon what happens between two time points cannot be made. This problem becomes obvious when unequally sampled data are used (Additional file 1, Figure S8).

Although the interpolation at the beginning of the time series (where the time points are rather close) seems to be sufficient, it does not accurately capture the dynamics of the expression time series when the distance between the time points becomes larger. Hence, by interpolating the gene expression data sets, artifacts are introduced, which will be further reflected in the results of the particular measures of interdependency. In order to avoid these artifacts, we renounce the interpolation in this comparison study, even though this leads to less significant results for almost all measures, as they operate far below the limit of their theoretically defined preconditions. However, we have observed that the overall results (*ROC *analysis) are typically equal or even better when interpolation is not included, especially when non-uniformly sampled time series are considered. Additional file 1, Figure S9 illustrates this effect exemplary for the simple mutual information.

However, some measures, such as the Granger causality used in this study, as well as several scoring schemes (*e.g.*, the Time Shift), are explicitly time dependent. Hence, they require uniformly sampled data, meaning that an interpolation is needed if only non-uniformly sampled data is available. This is in general, the case in *GRN *reconstruction.

However, most of the well performing reconstruction tools in our study are not explicitly time-dependent, which means they do not require a specific time sampling. This implicates that they are not very sensitive concerning the spacing on the time axis. Our results, as shown in Additional file 1, Figure S10, illustrate that a non-uniform sampling for these tools can even improve the quality of the reconstruction, since a larger period of the dynamics is captured.

### The role of the network topology

**Figure 4:**
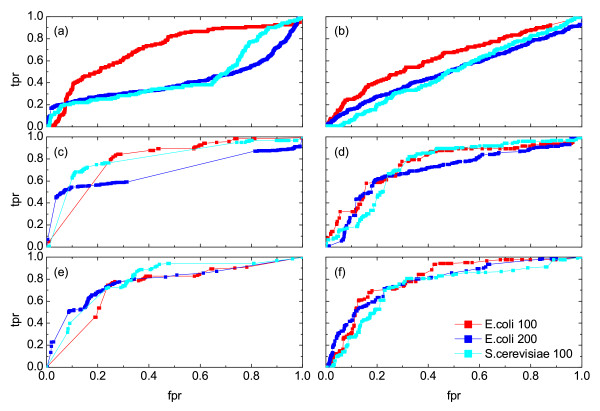
***ROC *curves obtained from the reconstruction of different networks**. The results are shown for an *E. coli *network of 100 genes, a *S.cerevisiae *network of 100 gene and an *E. coli *network of 200 genes using various similarity measures: (a) partial Pearson correlation , (b) conditional Granger causality , (c) Spearman correlation *μ_S_*, (d) simple mutual information *μ_I_*, (e) symbol sequence similarity , and (f) residual mutual information .

In general, the underlying network and its properties are not known prior to the reconstruction process. However, the available experimental and theoretical research has suggested that gene regulatory networks most likely are characterized with scale-free properties [39]. Therefore, we compare the reconstruction efficiency of the relevance network approach for various subnetworks of *E. coli's *and *S.cerevisiae's *regulatory networks, as described in details in the Methods section (subsection on Synthetic data sets). These networks (Additional file 1, Figure S14) differ in size, average degree and clustering coefficient. Nevertheless, we observe that the performance of the top-ranking measures, such as the symbol based measures, rank correlations, *MI *and *RMI *do not depend on the network topology: Very similar *ROC *curves are obtained for all of the network types analyzed, as shown in Figure 4 (this also pertains for several other measures, as shown in Additional file 1, Figure S11). The performance in the range of low *fpr *was improved for most of the measures for increased average degree of the nodes. However, at the same time, the performance in the range of high *fpr *was usually decreased. In general, the largest differences in the reconstruction efficiency occur for the conditional Granger causality and partial Pearson correlation, where the quality of the reconstruction decreases significantly for an increased number of nodes (*e.g.*, *E. coli *network with 200 nodes) and an increased clustering coefficient, as in the *S.cerevisae *network.

## Discussion

The observation of the *ROC *curves does not always allow conclusions on the overall performance of a measure or scoring scheme. Therefore, we further compare the described modifications of the relevance network algorithm in terms of *ROC *statistics (we focus on the algorithm as illustrated in Algorithm 1, without consideration of additional statistical significance due to the lack of a suitable null model). As an example, we evaluate the *ROC *statistics from time series with uniform sampling (without performing an interpolation) for the network of 100 genes of *E. coli*.

### ROC statistics for noise-free data

To evaluate and rank the overall performance of all approaches under study we calculate three common summary statistics from *ROC *analysis: the area under the *ROC *curve (*AUC*(*ROC*)), the *Y ouden *index and the area under the Precision/Recall curve (*AUC*(*PvsR*)) as explained in the Methods section. Furthermore, as the modifications of the algorithm implemented in the "minet" package are commonly and widely used approaches for *GRN *reconstruction, we use the results which gave the best performance in order to establish a benchmark for the comparison of the different measures and scoring schemes. In Table 2 we provide an overview of the results from the summary statistics for the different measures (mutual information and correlation estimation), and scoring schemes implemented in the *R*-package "minet". Based on these results, we define a measure combined with a particular scoring scheme to be

**Table 2:** The minet algorithm

PARAMETER (MINET)	*AUC*(*ROC*)	*YOUDEN*	*AUC(PvsR)*
clr, mi.empirical, equalfreq	0.80	0.54	0.05
clr, mi.empirical, equalwidth	0.76	0.45	0.04
clr, mi.mm, equalfreq	0.80	0.54	0.05
clr, mi.mm, equalwidth	0.76	0.48	0.04
clr, mi.shrink, equalfreq	0.80	0.53	0.05
clr, mi.shrink, equalwidth	0.74	0.41	0.04
clr, mi.sg, equalfreq	0.80	0.54	0.05
clr, mi.sg, equalwidth	0.74	0.42	0.04
clr, pearson, none	0.78	0.49	0.05
clr, spearman, none	0.80	0.53	0.05
clr, kendall, none	0.80	0.53	0.05
mrnet, mi.empirical, equalfreq	0.82	0.59	0.04
mrnet, mi.empirical, equalwidth	0.76	0.47	0.05
mrnet, mi.mm, equalfreq	0.81	0.57	0.04
mrnet, mi.mm, equalwidth	0.77	0.46	0.05
mrnet, mi.shrink, equalfreq	0.81	0.57	0.04
mrnet, mi.shrink, equalwidth	0.73	0.39	0.04
mrnet, mi.sg, equalfreq	0.81	0.57	0.04
mrnet, mi.sg, equalwidth	0.77	0.47	0.06
mrnet, pearson, none	0.78	0.49	0.04
mrnet, spearman, none	0.82	0.58	0.03
mrnet, kendall, none	0.81	0.56	0.03
aracne, mi.empirical, equalfreq	0.76	0.52	0.01
aracne, mi.empirical, equalwidth	0.54	0.12	0.02
aracne, mi.mm, equalfreq	0.76	0.52	0.01
aracne, mi.mm, equalwidth	0.54	0.12	0.02
aracne, mi.shrink, equalfreq	0.76	0.52	0.01
aracne, mi.shrink, equalwidth	0.55	0.14	0.02
aracne, mi.sg, equalfreq	0.76	0.52	0.01
aracne, mi.sg, equalwidth	0.54	0.12	0.02
aracne, pearson, none	0.54	0.07	0.03
aracne, spearman, none	0.76	0.52	0.01
aracne, kendall, none	0.76	0.52	0.01

Overview on the results of the summary statistics from the *ROC *analysis for the different parameter (implemented scoring schemes and measures for noise level 0.0) in the minet package.

• well performing for short expression data sets (evaluated on the synthetic data in this case) if:

*AUC*(*ROC*) > 0.8,

*YOUDEN *> 0.5 and

*AUC*(*PvsR*) > 0.05,

• sufficiently performing if

0.8 >*AUC*(*ROC*) > 0.7,

0.5 >*YOUDEN *> 0.4 and

0.05 >*AUC*(*PvsR*) > 0.03

• and deficient otherwise.

**Figure 5:**
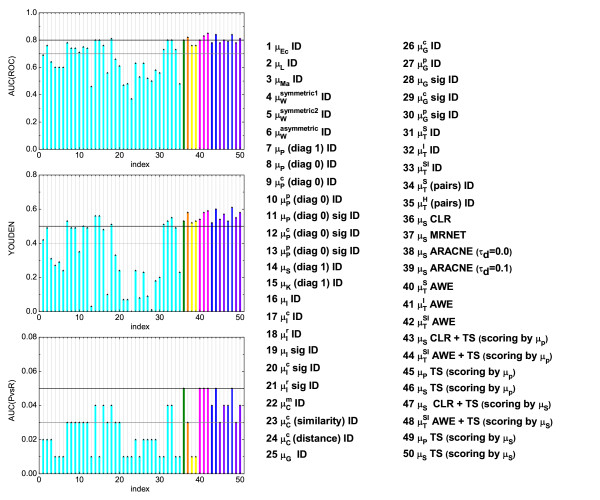
**Evaluation of the investigated scoring schemes/measures using the three different summary statistics (noise-free case)**. Similar approaches are grouped together. The first group in cyan refers to the different measures applied together with the *ID *scoring scheme. The green stands for the *CLR *scoring scheme, the orange for the *MRNET*, yellow refers to the *ARACNE*, magenta to the *AWE *and violet stands for the *TS*. These colors are related to those in Fig. 1. Furthermore, blue groups together all measures applied with a combination of scoring schemes.

By calculating the summary statistics in the noise-free case, as shown in Figure 5, we conclude that several information-theoretic measures (simple and residual *MI*), correlations (simple and conditional Pearson's, as well as Spearman's) and measures based on symbolic dynamics (*SyMI *and *SySimMI*) perform sufficiently well in combination with the basic relevance network algorithm (IDentity scoring scheme). Here, it stands out that the simple Spearman correlation performs better than the simple Pearson correlation, and *SyMI *is better than the simple *MI*. This is due to the fact that symbol and rank based measures are less sensitive to finite size effects and the distribution of data.

The modifications of the relevance network algorithm in the "minet" package having best performance in the reconstruction of *GRN *from short data sets, are the *CLR *and the *MRNET *("minet" is based on Spearman's correlation in this case). Here the *AUC*(*ROC*) indicates almost no change compared to the basic algorithm with identity scoring (measure: Spearman's correlation), while the *Y OUDEN *index decreases for the *CLR *and increases for the *MRNET*. However, the opposite is true for the *AUC*(*PvsR*). The overall performance of the *CLR *(in terms of the considered summary statistics) is slightly better than those of the *MRNET *(*CLR *scoring scheme was used to set the benchmarks). Moreover, the measures combined with the *TS *scoring scheme perform sufficiently well. However, the summary statistics do not change much compared to the results obtained for the same measures using the *ID*. In contrast, the asymmetric weighting yields a significant increase among all the summary statistics compared to the performance of the same measures using only the identity scoring scheme.

Hence, in the noise-free case, we obtain the following ranking of measures with the highest capability to detect true and eliminate false positive links:

1. *AWE *+ *TS *(scoring by *μ_S_*),

2. *AWE *+ *TS *(scoring by *μ_P_*),

3. *AWE*,

4. *AWE*,

5. *AWE *and

6. *μ_S _CLR*

The asymmetric weighting (*AWE*) significantly improves the prediction at this point, since it breaks the symmetry of a particular measure based on topological consideration and, therefore, reduces the number of false positive links. Hence the *AWE *(measure: ) clearly shows the best performance when short time series are considered (the results become slightly better if Time Shift is applied in addition).

### ROC statistics for noisy data

**Figure 6:**
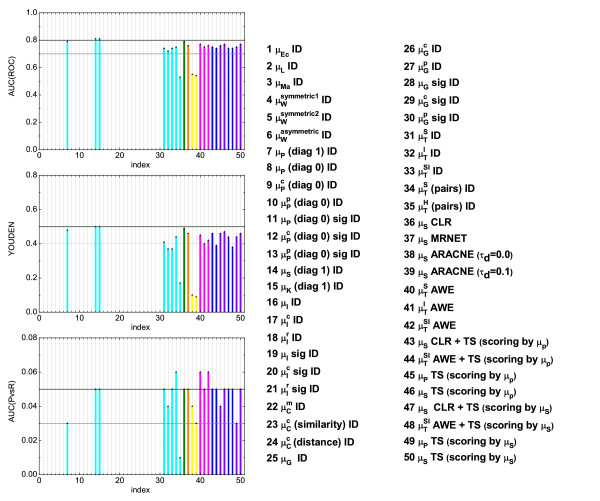
**Summary statistics considering moderate noise (noise level 0.3)**. The results for selected measures using different scoring schemes are shown. Similar approaches are grouped together here in the same way as in Fig. 5.

In order to account for stochasticity in the time series as well as to establish the robustness of the investigated (top-ranked) similarity measures against noise, we evaluate additionally their performance for two different noise intensities, namely 0.3 (Figure 6) and 0.5 (Additional file 1, Figure S12). Only those measures which perform sufficiently well in the noise-free case (measures operating on random variables and symbolic dynamics) are tested. In particular, we examine the Pearson's (*μ_P_*(*x*, *y*)), Spearman's (*μ_S_*(*x*, *y*)) and Kendall's (*μ_K_*(*x*, *y*)) correlation coefficients as well as the symbol based measures , , , and  using the *ID *scoring scheme. Additionally, we investigate the performance of *CLR*, *MRNET*, *ARACNE*, *AWE *and *TS *scoring schemes based on the same measures as in the noise-free case.

Under the influence of noise, the quality of the results of the symbol based measures (in particular ) decreases. As noise strongly influences the process of symbol assigning, it can principally enhance or distort the information content. The direction of the influence is not predictable *a priori*, but in the presence of strong noise, symbols are no longer reliable (if no additional information on the influence of the noise is provided). On the other hand, measures operating on random variables are rather robust against noise (the best results in these cases have been achieved using rank correlations).

The *ARACNE *has proven to be very sensitive with respect to noise. In contrast to this, the asymmetric weighting (compared to the results of the *ID *using the same measure) still performs well within the given limits, as it is only based on topological considerations, and it is not influenced by the presence of noise. Furthermore, to investigate how noise influences the reconstruction efficiency, we calculated the area under the *ROC *curve and the *YOUDEN *index as a function of the noise intensity for the 5 combinations of similarity measures and scoring schemes which performed best in the noise-free case, namely the symbolic measures and the asymmetric scoring schemes, mentioned in the previous section. Additionally, we compared the results to those obtained for time series of different lengths (*i.e.*, 8 and 20 time points). We conclude that for short time series, the capability of the measures and scoring schemes to detect true and at the same time eliminate false positive links depends both on the number of time points and the noise intensity (Additional file 1, Figure S13). However, this dependence is small compared to the differences in the reconstruction efficiency between the various measures. Moreover, the sensitivity against noise is reduced with increased length of the time series (which corresponds to the usage of order pattern of higher dimension). In general, we observe a decrease in the reconstruction efficiency if the noise levels increase or the length of the time series decreases. For the short time series used in this study, however, these dependencies are not monotone.

## Conclusions

By performing an extensive comparison analysis of the reconstruction efficiency of the relevance network algorithm using 6 scoring schemes and 21 different measures, we showed that with a suitable choice of a measure and a scoring scheme, this approach is applicable to short time series to gain knowledge about the underlying gene regulatory networks which differ in various properties. However, most of the currently used measures have highly limited capabilities, as the number of time points of the gene expression data is usually not sufficient to infer the underlying structure of the network. This in turn make the distinction between direct and indirect interactions an even more challenging task.

This study could serve as a basis for the selection of a reverse engineering method for network reconstruction, based on the combination of a similarity measure and a scoring scheme suitable for given data. Our results showed that rank and symbol based measures (which we applied for the first time for *GRN *reconstruction) have a significantly better performance in inferring interdependencies, whereas most of the standard measures (such as Granger causality and several information-theoretic measures) fail when short time series are considered. The residual mutual information, which we proposed in this work as a partial mutual information measure, increased the reconstruction efficiency of the relevance network algorithm compared to simple and, in particular, conditional mutual information.

Nevertheless, from the analysis presented here, we conclude that it is necessary to move further from the standard similarity measures based on the time series directly, towards measures rooted in the study of symbolic dynamics or ranks deduced from the time series, in order to increase the efficiency of the relevance network algorithm for *GRN *reconstruction. Although measures based on symbolic dynamics performed significantly well in the noise-free case, their performance was decreased as the noise level in the system increased, and for high noise intensities it became comparable to that of mutual information. This implied that in the presence of strong noise, rank correlations (in particular Spearman's rank correlation) are most efficient tools for *GRN *reconstruction, since their performance was not significantly affected as the noise level increased. Additionally, we note that the results obtained for *RMI*, rank correlations and symbol based measures are robust with respect to the network topology. We also showed that an unequal sampling of the data in general does not pose additional problems if measures are considered where interpolation is not essential (such as the top-ranked measures in this study).

We point once again that all rank and symbol based measures described here are symmetric. This means that the directionality of the interactions cannot be inferred, unless a symmetry-breaking scoring scheme is considered in addition. In that direction, we showed that a novel scoring scheme, the asymmetric weighting (*AWE*), which we proposed in this work stands as a valuable approach to overcome the problems of introducing directionality in the reconstruction of the regulatory networks.

It would be interesting to compare in future the observed reconstruction efficiency of the relevance network approach to that of other reverse engineering methods, such as the Bayesian network approach.

## Methods

Our work focuses on methods for reverse engineering which operate on time-resolved gene expression experiments (in terms of mRNA concentrations). We define a time series profile for a gene measured over *n *time points as a sequence of expression values *x *=<*x*_1 _, ..., *x_n _*>, where each *x_i_*, 1 ≤ *i *≤ *n*, corresponds to a distinct time point *t_i_*. In addition, let each of the *m *genes be represented by *r *time-resolved replicates over *n *time points. Here, we use the mean of *r *= 6 replicates, resulting in an *m *× *n *data matrix *M*. Let *M_i_*, 1 ≤ *i *≤ *m*, denote the *i^th ^*row of a matrix *M*, which corresponds to the time-resolved expression profile of the *i^th ^*gene.

The general reverse engineering method based on a particular similarity measure *μ *and a scoring scheme *F *operating on the data matrix *M *is given in Algorithm 1.

**Input**:

*M*, matrix with *m *rows (genes) and *n *columns (time points),

*μ*, similarity measure,

*F*, scoring scheme

**Output**:

*m *× *m *adjacency matrix, *A*, of the reconstructed network *G*

**1 foreach ***gene i, i *∈ {1,..., *m*} **do**

**2   foreach ***gene j*, *j *∈{1,..., *m*}, *j *≠ *i ***do**

**3**      *w_ij _←|μ*(*M_i_*, *M_j_*) *|*

4   end

5 end

**6 ***C *: *c_ij _← w_ij _· f_ij _*;

**7 **chose a threshold *τ *;

**8 ***a_ij _← *1 if *c_ij _*>*τ*;

**9 ***a_ij _← *0 if *c_ij _*≤ τ;

**Algorithm 1**: General reverse engineering method based on a similarity measure *μ *and a scoring scheme *F*. *f_ij _*∈ *F*, *c_ij _*∈ *C*, *a_ij _*∈ *A*, and *w_ij _*∈ *W*, where *W *is the matrix obtained by applying *μ *on all pairs of rows of the given data matrix *M*.

The evaluation of the scoring schemes and measures is generally performed in *R *[40] using available packages, as noted in the manuscript. Additionally, several *C *routines were developed in order to improve computational speed.

In what follows, we describe the procedure for generating the synthetic time series data, and present the definitions of the similarity measures and scoring schemes used in the comparative analysis. Furthermore, the details of the *ROC *analysis are briefly reviewed.

### Synthetic data sets

The evaluation of the existing methods for reverse engineering gene regulatory networks often employs real time-resolved expression data. However, these data include the convoluted effects of regulons (genes under regulation by the same regulatory protein) and stimulons (genes under regulation by the same external influence), which renders it challenging to realistically assess the performance of investigated methods. Moreover, not every regulatory subnetwork leads to expression of the participating genes over the measured time period and particular condition of interest. These facts lead to a lack of control when using transcriptomics time series data sets for network inference.

Following the example of the DREAM challenge [2,14], in this comparison study we use synthetically generated data sets to overcome the described disadvantages. The usage of these synthetic data, in contrast to real measurements, enables us to directly compare the performance of different reconstruction tools, since the topology and dynamic of the underlying network is known *a priori*. In particular, we use the synthetic network generator, SynTReN [41,42], which creates synthetic transcriptional regulatory networks by providing the edges of the network, as well as information about the interaction (activating, repressing, or dual). Additionally, the generator produces simulated gene expression data of the associated mRNA concentrations for each gene, based on Michaelis-Menten and Hill kinetics, which approximates experimental expression measurements. These expression data sets are uniformly sampled in time.

In SynTReN, the levels for three types of noise are user definable: (1) biological noise, corresponding to biological variability given by the stochastic variations in gene expression, (2) experimental noise, corresponding to the technical variability, and (3) noise on correlated inputs, which accounts for the influence of several activated genes on a regulated gene. Note that different noise levels are included in this study, but we make no distinction between the strength of the three noise types.

**Figure 7:**
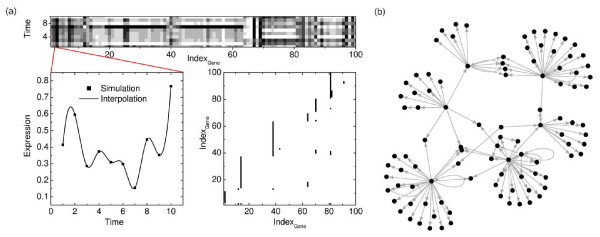
**Test data set for the comparison study**. (a) The *GRN *of *m *= 100 genes in *E. coli *is illustrated in the lower right panel as an adjacency matrix. Each entry marks a regulatory link between two associated genes. The upper panel shows the corresponding expression time series (simulated in the noise-free case and normalized to values between 0 (coded in black) and 1 (coded in white)). An example of the time series of the *lon *gene (gene number 2), including a spline interpolation is shown in the lower left panel. (b) The graphical representation of the network is shown in addition.

In particular, we generate regulatory networks from the *GRN *of *E. coli *and *S. cerevisiae*, using the cluster addition strategy to select a connected subgraph: In each iteration, a node is randomly chosen and added to the graph together with all its neighbors. This strategy is chosen since it is an efficient method to extract a subnetwork that approximates well the topology of the source network. The results presented in this work are obtained for subnetworks of distinct sizes which differ in degree and clustering coefficient. In particular, we investigate an *E. coli *subnetwork of 100 genes including 121 links where 10 of the genes code for transcription factors. It is characterized by an average degree of 2.42 and a clustering coefficient of 0.016. Additionally, we examine two more networks: (1) An *E. coli *subnetwork of 200 genes (34 coding for transcription factors) that includes 303 links and is characterized by an average degree of 3.03 and a clustering coefficient of 0.019, and (2) a *S. cerevisiae *subnetwork of 100 genes (14 coding for transcription factors) that includes 123 links and is characterized by an average degree of 2.46 and a clustering coefficient of 0.026. The degree distributions are shown in Additional file 1, Figure S14. Synthetic gene expression data with 10 biological (*n *= 10 time points) and 6 technical (*r *= 6) replicates have been generated from the particular networks. We use the means of the technical replicates for the interaction analysis. An example of a generated network (100 in *E. coli *used for our investigations), and the simulated gene expression data sets are visualized in Figure 7.

### Interpolation

Since transcriptomics time series data usually consist of expression at a few, possibly non-uniformly sampled time points, data interpolation is often the first pre-processing step. Different techniques, including linear [43] and nonlinear interpolation methods, such as cubic- [33] and b-splines [25] have been applied [11]. Although these methods extend the available data, they also introduce artefacts due to over-fitting. For this reasons, we preclude from interpolating the synthetic time series data (whenever possible) for ranking of the used similarity measures. Nevertheless, for reason of comprehensiveness, we consider the effect of interpolation using cubic splines on the investigated similarity measures and scoring schemes of highest rank in absence of noise.

### Similarity measures

Reverse engineering of regulatory networks relies on the inference of interrelationships among genes, based on similarity measures. In general, given two time series *x *and *y *over *n *time points, a *similarity measure *is given by the mapping *μ *: **R^n ^**× **R^n ^**→ *I*, where *I*, *I *⊆ **R**. The so-defined pairwise similarity measure detects (non)linear relationships between two variables, in the considered case, between two gene expression time series. The definition allows for the measure to be symmetric, which is not commonly the case for gene regulatory interactions. Moreover, if two genes are linked indirectly via a third gene, the pairwise measure can not distinguish direct from the indirect relationships and hence additional false positive links will be introduced in the network reconstruction.

Nevertheless, the definition of the similarity measure can be extended to conditional and partial measures, incorporating the possibility to exclude the influence of a third gene. Conditional similarity measures are more general, since they do not rely on specific assumptions on the probability distribution (deduced from the time series associated with a discrete random variable), but estimate the distribution which in turn impedes the computation of the measure from short time series. On the other hand, partial measures can indicated conditional independence reliable only for multivariate Gaussian variables.

To be able to discern the direction of a putative interaction and hopefully eliminate any spurious effects, we consider the conditional and partial variants for several of the measures detailed below. We term the basic pairwise measures as simple, in comparison to their conditional and partial variants. An overview on the measures included in this study is given in Table 1.

#### Measures operating on vectors

Some of the standard measures used for determining gene regulatory interactions are based on the calculation of the distance between expression time series regarded as vectors. In the following, *x *and *y *will denote the vectors <*x*_1_, ..., *x_n _*> and <*y*_1_, ..., *y_n _*>, respectively. Our study includes:

##### *L^s^*** norm: **

This distance measure for vectors *x *and *y *is defined as follows:(1)

In our study, *s *= 10, which corresponds to the number of available time points.

##### Euclidean distance

Furthermore, we consider the well-known Euclidian distance, which is a special case of the *L^s ^*norm, with *s *= 2. Therefore, it is defined as(2)

##### Manhattan distance

We also study the performance of the Manhattan distance which represents the shortest path between two points, placed on a rectangular grid, and is analogous to the *L*^1 ^norm:(3)

##### Dynamic time warping (*DTW*)

**Figure 8:**
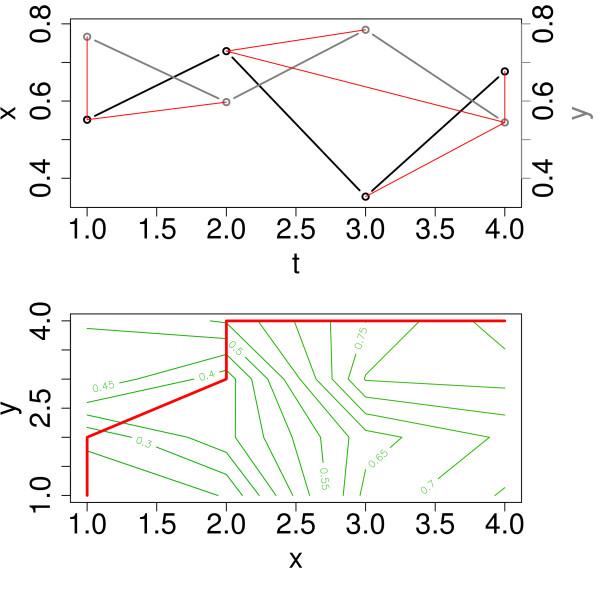
**Illustration of the concept of dynamic time warping (*DTW*)**. The upper panel shows two time series *x *(black) and *y *(gray), as well as a mapping (red lines) of the time points in *x *into those in *y*. This mapping is optimal with respect to the step pattern "symmetric2", meaning the sum of all incorporated local distances (represented by lengths of the red lines) is minimal, given the constraints from the step pattern. The lower panel shows all local distances between time points in *x *and *y *in a contour plot, where the red path is associated with the lowest value of the cumulative distance (optimal alignment path).

In addition, we investigate the performance of the *DTW*, which to our knowledge, has not been applied to the problem of gene regulatory network inference, but rather on clustering genes expression data [43,44]. The *DTW*-based measure relies on finding the optimal (least cumulative) distance mapping a given time series into a reference time series, where both sequences may vary in time and/or speed. It was originally developed for speech recognition [45,46], but has been recently used for different data mining tasks in medicine and bioinformatics [43,47]. The concept of *DTW *is sketched in Figure 8 for two short time series with 4 time points each. In the first step of the *DTW *algorithm, local distances (*e.g.*, Euclidean or Manhattan distance) for all pairs of time points are calculated. Then, the time series are mapped into each other by linking various time points, such that each point is included at least once and the sum over the lengths of all those links is minimal (optimal alignment path). Here, we use the *DTW *as implemented in the *R*-package "*d*tw" [48-50], with the Euclidean as point-wise local distance, and different step patterns which indicate the local constraints of the alignment paths. We include three different step patterns, namely

• symmetric1(4)

• symmetric2(5)

• and asymmetric(6)

to find an optimal alignment. Here *μ_EC _*denotes the local (Euclidean) distance, and the measure *μ_W _*the cumulative distance (representing the minimum sum of local distances along the alignment paths).

The resulting matrix of cross-distances *D *contains the pairwise calculated distance measures (*μ_EC_*, *μ_L_*, *μ_MA_*, or *μ_W_*, as defined above) and is, in all cases, normalized by the largest value occurring in the matrix as follows:(7)

The similarity measure is then defined by:(8)

#### Measures operating on random variables

Despite the representation of the expression time series as vectors, time series *x *= <*x*_1_, ..., *x_n _*> can be associated with a discrete random variable *X *with probability distribution *p*(*x*), *x *∈ *X *that is approximated by the frequency via standard binning arguments. This representation allows to calculate several widely used similarity measures, such as correlation and information-theoretic measures. Note that the temporal information is lost by this representation of time series data. We first review the most commonly used measures in detail:

##### Pearson correlation

This similarity measure quantifies the linear relationship between the random variables *X *and *Y*, corresponding to two time series *x *and *y*:(9)

where *E *denotes expectation(10)

If the variables are independent, the correlation coefficient is *μ_p _*= 0, but the opposite is not true, as this coefficient is sensitive mainly to linear dependencies. Note that *μ_P _*receives values in the interval [-1, 1] and is symmetric.

##### Conditional Pearson correlation (*CPC*)

By using the conditional expectation value(11)

where *p*(*x|y*), *x *∈ *X*, *y *∈ *Y *is the conditional probability distribution, one can provide the following definition for *CPC*:(12)

Thus, the conditional correlation among the time series *x *and *y *of the corresponding genes, eliminating the influence of all other genes is defined as(13)

##### Partial Pearson correlation

Analogously, one could also consider(14)

where(15)

The residuals are calculated following Eq. (16) making a linear regression of *x *(respectively *y*) depending on *z*:(16)

Rank correlations can be used as a more general measure of interdependence, not restricted to a linear relationship. Even though they measure a different type of relationship than the product moment correlation coefficient, like the previous correlation measures, these are also defined in the interval [-1, 1].

##### Spearman's rank correlation

This type of correlation is based on the rank distribution of the expression values:(17)

where *R*(*x*) is the rank of *x*.

##### Kendall's rank correlation

Another rank correlation is(18)

with *n_c _*being the number of concordant pairs, and *n_d _*the number of discordant pairs of the rank sets.

It is common to regard the rank correlation coefficients (especially Spearman's rank correlation) as alternatives to Pearson's coefficient, since they could either reduce the amount of calculation or make the coefficient less sensitive to non-normality of distributions. Nevertheless, they quantify different types of association.

Unlike most of the measures discussed here, the correlation measures do not only provide an information about whether two genes are interacting, but also whether it is an activating or repressing relationship. As the latter information is outside of the interest of the current study, only the absolute value (respectively the square) of the correlation coefficient is taken into account.

Additionally to the correlation, information-theoretic measures are also defined using random variables as relevant representation for expression time series.

##### Mutual information

One of the most commonly used measures for inferring interdependencies between two subunits of a system is the mutual information (*MI*) [16]. Intuitively, *MI *measures the information content that two random variables *X *and *Y *share. The **simple mutual information **can then be expressed in terms of the marginal entropies *H*(*X*) and *H*(*Y *), and the joint entropy *H*(*X*, *Y *) using the definition of the Shannon entropy(19)

which quantifies the uncertainty associated with a random variable. Hence the simple *MI *is defined as(20)

It includes also non-linear interrelations, but same as the other simple measures, the simple *MI *cannot be used to distinguish between direct and indirect relations.

##### Conditional mutual information

However, if we replace the marginal and joint entropies by the conditional analogs, *H*(*X|Z*), *H*(*Y|Z*) and *H*(*X*, *Y|Z*), we can eliminate the influence of a third variable *Z*. Hence, we calculate the minimal information shared by the time series *x *and *y *of two genes conditioned on each *z_k_*, *k *= 1, ..., *m*. Thus the **conditional**--sometimes also referred to as partial [51]--**mutual information **(*CMI*) can be written as:(21)

Where(22)

The degree of interaction is indicated by the values of *μ_I _*or  normalized by the largest value occurring among all pairs of genes.

##### Mutual/conditional coarse-grained information rate

Another approach based on information-theoretic aspects are the coarse-grained measures. Here, instead of approximating the exact entropies of time series, relative measures of "information creation" are used to study the interrelationship of two (sub)systems. Thus, for this purpose, the calculation of coarse-grained entropy rates [52] is used to replace the approximation of the Kolmogorov-Sinai entropy (metric entropy of a dynamical system): First, a time lag *l_max _*is determined such that(23)

among all analyzed data sets. Then, the coarse-grained information rate (*CIR*) is given by the norm of the mutual information(24)

Usually the parameter *l_min _*and Δ*l *(difference between consecutive time lags) can be set to one, and thus the *CIR *becomes(25)

Hence, the **mutual coarse-grained information rate **(*MCIR*) is defined as(26)

whereas the **conditional coarse-grained information rate **(*CCIR*) as(27)

with(28)

Finally, a normalization by the largest value occurring among all pairs of genes is performed. These (normalized) coarse-grained information rates are then used to indicate the degree of interaction.

#### Model-based measures

##### Granger causality

A rather new approach for inferring gene regulatory networks is the Granger causality (*GC*). Given the time series *x *and *y*, two linear autoregressive (*AR*) models are estimated, both including the past of *x*, and additionally, one of them including the past of *y*. In order to determine the optimal order *q *of the *AR *model, which denotes the number of past time points which have to be included, we use the function "*VARselect*" from the *R*-package "*vars*" [53,54] based on the Akaike information criterion (*AIC*) [55]. The *AIC *is a measure of the goodness of a fit of an estimated statistical model, deduced as a tool for model selection. In the general case, the *AIC *is defined as:(29)

where *u *is the number of parameters in the statistical model, and *L *is the maximized value of the likelihood function for the estimated model.

With properly selected *AR *models, the part of the variance in the data which is explained by one model in comparison to the other one, provides an information on the causal relationship. This comparison can be formulated in terms of an index.

Thus the **Granger Causality index**, denoted by *μ_G _*for the simple linear measure, as defined in [34,35] via the covariance *σ*, is:(30)

and can be inferred from the *AR *models:(31)(32)

where *a*_11*i*_, *a*_21*i *_and *a*_22*i *_are the parameters of the models and *u*_1*t*_, respectively *u*_2*t *_represents white noise.

##### Conditional/partial Granger causality

As for the previous measures, we use the conditional and partial (linear) Granger causality measures (*CGC *and *PGC*) as defined in [34,35], in order to identify existing indirect relationships. Hence, the *AR *models are formulated as(33)(34)

for the conditional, and, in addition,(35)(36)

for the partial Granger causality, with *a*_11*i*_, *a*_12*i*_, *a*_21*i*_, *a*_22*i*_, *a*_23*i*_, *a*_31*i*_, *a*_32*i*_, *a*_41*i*_, *a*_42*i *_and *a*_43*i *_being the parameter of the models and *u*_1*t*_, *u*_2*t*_, *u*_3*t *_and *u*_4*t *_representing noise terms.

Using the Eqs. (33) and (34), the **conditional Granger causality index **is then defined as:(37)

where(38)

and, using the Eqs. (33) to (36), the **partial Granger causality index **is(39)

where(40)

The degree of interaction is indicated by the Granger causality index normalized by the largest value occurring among all pairs of genes.

#### Measures operating on symbolic dynamics

Despite the promising applications of interaction measures based on symbolic dynamics in various fields, they have not yet been employed for reverse engineering gene regulatory networks. For instance, in standard nonlinear time series analysis, the usage of symbolic dynamics to uncover patterns of interactions, especially from short data sets [56], has proven as a valuable tool. Therefore, we explore the potential of symbolic dynamics for the problem at hand by using the principle of order patterns. By this principle, as described in [38], the time series are transformed into symbol sequences. An order pattern *π *of dimension *δ *is defined by the discrete order sequence of the time series *x *and has the length *δ*. Hence, the time series can be symbolized using order patterns following:(41)

where *l *is the time lag. In terms of gene regulatory network reconstruction, we need to choose a certain number of time points and rank them according to their expression value in order to obtain the order pattern. Then, each possible ordering corresponds to a predefined symbol.

**Figure 9:**
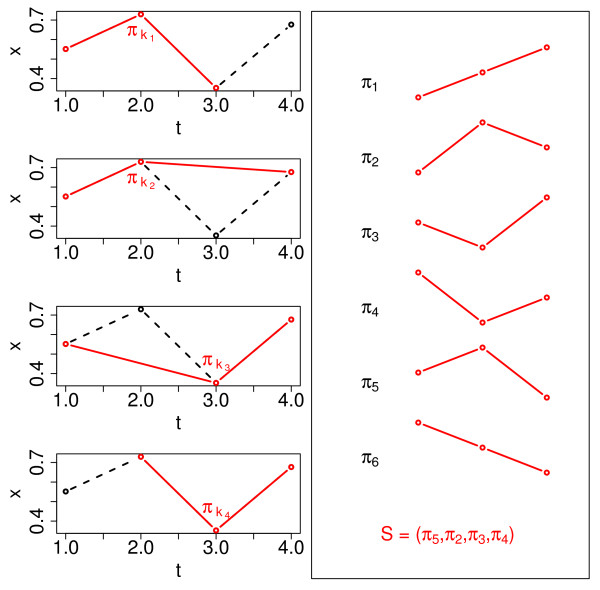
**Illustration of the concept of order pattern**. The left panels show a time series (black) composed of *n *= 4 time points and particular groups of 3 time points each which are forming order pattern of dimension *δ *= 3 (red). The possible order pattern of that dimension are overviewed in the right panel together with the resulting symbol sequence *S *for the mentioned time series.

This concept is illustrated in Figure 9 for time series composed of *n *= 4 time points. As we are dealing with very short time series here, we consider all possible combinations of the chosen number of time points. For instance, for the time series of length *n *= 4 and an order pattern of dimension *δ *= 3, we define symbols (order patterns *π_k_*) for the following groups of time points: (1, 2, 3), (1, 2, 4), (1, 3, 4) and (2, 3, 4), shown in the left panels of Figure 9. Next, we define a symbol sequence(42)

where  denotes the order pattern obtained for gene *i *from the *k*-th group of time points and(43)

is the length of the symbol sequence.

In this work, we usually choose the dimension *δ *such that the length *T *becomes maximal, given a time series of length *n *(*i.e.*, *δ *= 5 for *n *= 10). However, if the symbol sequences are calculated for longer time series, this approach is not applicable anymore. This is due to the fact that calculations in *R *are not possible because of the fast growing length of the symbol vectors. Hence, we use for the time series of 20 time points order pattern of dimension 6 instead of the dimension 10, which would lead to the symbol vector of maximal length.

##### Symbol sequence similarity

Using the above described approach, we infer the interdependency of two genes as follows: Given a certain number *δ *of time points we define a vector *P *containing all possible permutations of the ranking, and assign a symbol (order pattern *π_k_*) to each of them. Next, we define a vector , using the same symbols as for *P*, but assigned to the reversed ranking. Now, we count the pattern overlap of two symbol sequences *S*^(*i*) ^and *S*^(*j*) ^to evaluate the **symbol sequence similarity**, *p*_1_, assuming both time series are interrelated (Eq. (44)), respectively *p*_2 _if we assume anti-interrelation (Eq. (45)).(44)(45)

We choose the maximal value of the two frequencies *p*_1 _and *p*_2_(46)

to define the symbol sequence similarity (*SySim*).

##### Mutual information of symbol vectors

We calculate the **mutual information of the symbol vectors **(*SymMI*) of maximal length by:(47)

In addition, we consider the mean of the **symbol sequence similarity and the mutual information **(of the symbol vectors) (*SymSimMI*)(48)

This is further extended to include symbolic dynamics based on a slope comparison (order patterns for pairs of time points), where we consider

• the **symbol sequence similarity for pairs **(*pairs*) as a similarity measure

• and the **conditional entropies for pairs **(*pairs*) as a distance measure, with .

#### A novel measure -- the residual mutual information

Estimating entropies from short time series is imprecise, hence the estimation of the mutual information and, in particular, its conditional counterpart, suffers the same disadvantage. On the other hand, the simple mutual information is not able to distinguish between direct and indirect links. Therefore, in order to overcome the encountered problem, we propose a novel partial measure -- the **residual mutual information **(*RMI*) defined as:(49)

where(50)

analogously to the idea of partial correlation (the residuals are calculated in the same way as for the partial correlation in Eq. (16)). The degree of interaction in the complex network is then indicated by the values of , normalized by the largest value occurring among all pairs of genes.

Applied to short data sets, we expect that the residual mutual information performs much better in discriminating indirect links than the conditional *MI*, as we can abandon the estimation of additional conditional probabilities. Hence, the measure is more robust to effects of small sample size. Furthermore, we expect that the *RMI*'s performance ranges between those of the simple and the conditional mutual information for long time series, since in contrast to the *CMI*, we eliminate here only the linear influence of the variable *Z *on *X *and *Y*. We postpone the confirmation of this claim for further theoretic analysis.

### Scoring schemes

Once a chosen similarity measure has been applied on a given data matrix, there are several possibilities to score the resulting "weights" of putative interactions. In this sense, a *scoring scheme F *is a matrix of dimensions *m *× *m*. Let *W *denote the matrix obtained by applying *μ *on all pairs of rows of a given data matrix *M*, for *w_ij _*≥ 0, ∀*i*, *j*. The scores from a given scoring scheme and similarity measure can then be represented by a matrix *C *calculated from the Hadamard element-wise product of *W *and *F*, such that *c_ij _*= *w_ij _*· *f_ij_*.

To unravel the linkage of genes we apply the relevance network algorithm (Algorithm 1) using different scoring schemes and measures. In principle, all of the measures can be combined with any scoring schemes, but we restrict our investigations to the most commonly used.

#### IDentity (ID)

The identity scoring scheme corresponds to the basic relevance network [26] approach: Given a specific measure, a particular threshold *τ *is set in order to account for "true" links between elements in the network. The matrix *F *is the unit matrix (*f_ij _*= 1) in this case. Therefore, for a symmetric similarity measure, the identity scoring scheme cannot infer directionality of interactions. We test the performance of all measures mentioned above in combination with this scoring scheme.

#### Context Likelihood of Relatedness (CLR)

As a second type of scoring scheme, often used for the reconstruction of *GRN*, we consider the *CLR*, which is an extension to the basic relevance network approach. Once weights *w_ij _*have been assigned for each pair of genes according to the strength of interaction inferred from a particular measure, a score is derived, related to the empirical distribution of the values in *W*. Thus, the matrix *F *obtains the form(51)(52)(53)

where  and *σ_i _*(*σ_j_*) are the mean and standard deviation of the empirical distribution of *w_ik _*(*w_jk_*), *k *= 1, ..., *m*. The links having *c_ij _*<*τ *(with *c_ij _*= *w_ij _· f_ij _*and *τ *a predefined threshold) are removed for the network reconstruction.

Here, we use the *CLR *as implemented in the *R*-package *"minet" *[57,58], which uses either the **simple mutual information **or a **squared correlation matrix **(Pearson's, Spearman's or Kendall's) to measure the strength of interaction among genes. We note that the *CLR *algorithm cannot infer directionality from symmetric measures.

#### Algorithm for the Reconstruction of Accurate Cellular Networks (ARACNE)

Furthermore, we investigate the Algorithm for the Reconstruction of Accurate Cellular NEtworks, referred to as *ARACNE*, and include its performance in the current comparison study. The *ARACNE *is based on the data processing inequality, which states that post-processing cannot increase the amount of information. Hence it follows that:(54)

when gene *i *and *k *are not directly linked, but this goes through *j*, where *x_i_*, *x_j _*and *x_k _*are the expression time series of these genes. In this manner, the algorithm discriminates indirect links. *ARACNE *is a relevance network algorithm as illustrated in Algorithm 1 as well. First, weights *w_ij _*(normalized to the interval [0, 1]) are assigned to each pair of nodes. Then the scoring scheme operates as follows: For each triplet of nodes the edge having the lowest weight will be removed (its weight is set to zero), if the difference between the two lowest weights is above a threshold *τ_d_*, as that interaction is interpreted as indirect. In this manner, the matrix *F *obtains the form:(55)

Moreover, the *ARACNE *removes all edges satisfying *c_ij _*<*τ*, where *τ *is a predefined threshold.

As for the *CLR *algorithm, we rely on the "*minet*"-package, using the **simple mutual information **or a **squared correlation matrix **to determine the weights. By default, the two thresholds are set to zero. The *ARACNE *does not distinguish the direction of a link from a symmetric measure as well.

#### Maximum Relevance/minimum redundancy NETwork (MRNET)

As another example of a relevance network algorithm, we consider the Maximum Relevance/minimum redundancy NETwork (*MRNET*) [59]. This scoring scheme performs series of supervised maximum relevance/minimum redundancy (*MRMR*) gene selection procedures, where the expression of each gene in turn plays the role of the target output *y *= *x_i_*, with *V *= *x*\*x_i _*being the set of the expression data of the input variables, and *x *the set of the expression levels of all genes. Given the set *M *of selected variables and pairwise weights *w_ij|M_*, the criterion updates *M *by choosing the variable(56)

that maximizes the score(57)

where  is a redundancy term, and *u_j _*= *w_ji _*is a relevance term. This scheme therefore assigns higher rank to direct interactions, whereas indirect interactions (redundant information with the direct ones) should receive lower rank. Thus, the matrix *F *is defined as:(58)(59)(60)

Finally, all edges whose score *c_ij _*lies below a predefined threshold *τ *will be removed.

The implementation of the *MRNET *in the "*minet*"-package, which we used in our study, assigns the weights based on the pairwise **simple mutual information **or a **squared correlation **among the time series of two genes (normalized to the largest value occurring among the pairs). Also this algorithm is not able to infer directionality from symmetric measures.

#### Time Shift (TS)

In nonlinear time series analysis, the shifting of time series is a common way to infer the directionality of causal relationships. As the driving system by definition has to act first, shifting its time series forward in time (relative to the time series of the response system) should increase the similarity of both time series. Comparing the values of a particular measure for different time shifts gives then a hint on the direction of the interaction. Thus, the time shift scoring scheme starts with a cubic spline interpolation for each pair of genes expression time series. Then the series of the second gene is shifted against that of the first gene. If *x *and *y *are two expression time series stored in the *i^th ^*and *j^th ^*row of the data matrix *M*, and  and  are the related interpolated time series, we can then define the shifted time series as(61)

and(62)

where *N *is the length of the interpolated time series and *N_shift _*is the assumed shift of  versus , with *N_shift _*∈ **Z **and *N_shift _*∈ [-0.1 · *N*, 0.1 · *N*]. Next,  is evaluated for all possible values of *N_shift_*, resulting in a vector  (for not significant values of *μ *the corresponding entry will be set equal 0). The scoring is now given by(63)(64)(65)

In case the largest significant value of the measure is obtained for a negative shift, the regulatory direction from the first to the second gene is kept, while the opposite direction is preserved if the largest significant value is obtained for a positive shift. Furthermore, both regulatory directions are kept, if the maximum arises for a shift of zero or multiple opposed shift values or in the case when no significant value exists. The scoring scheme aims at providing a hint on the directionality, because the absolute values of the calculated correlations on the delayed time series are rather biased as the data sets are quite short.

In the next step of Algorithm 1, the information regarding the directionality are combined with the weight of interaction inferred from a particular measure (*c_ij _*= *w_ij _· f_ij_*). Hence, the weights for the unlikely direction are set to zero in order to break symmetries, and thus reduce the number of false positives. Finally, all edges with *c_ij _*<*τ *are removed, where *τ *is a particular threshold.

We test that scoring scheme using the **absolute value of of the correlation coefficients ***μ_P _***(Pearson) **and *μ_S _***(Spearman) **for pairs of the shifted expression series, where the significance level was set to *α *= 0.01 and only absolute values of correlation larger 0.9 have been taken into account. The choice of the measure to infer the weights in the first step of Algorithm 1 is independent of that and includes here the **mean of sequence similarity and mutual information of symbols**, as well as **Spearman's **and **Pearson's correlation**. Furthermore, this scoring scheme is applied in addition to (or after) another scoring scheme (*e.g.*, *ID*, *CLR*, or *AWE*). It is important to note that in contrast to the previously described modifications of the algorithm, the scoring scheme we propose here allows to investigate the directionality, also when symmetric measures are considered.

#### A novel scoring scheme - Asymmetric WEighting (AWE)

Most of the measures used to infer the degree of interaction between pairs of genes, such as correlations or the mutual information, are symmetric. Hence, when applied in symmetric algorithms they are not able to unravel the regulatory dependences, since these measures do not distinguish the direction of the interaction. Thus, we introduce an asymmetric weighting based on topological aspects, for the complete set of pairwise weights obtained from a particular measure, and implement it according to Algorithm 1. In particular, we compute a matrix of weights, where the columns represent the genes which are regulated, and the rows stand for the genes which regulate other genes. The scoring value is then calculated by dividing each row entry by the sum of the corresponding column values. The scoring scheme (and the corresponding matrix *F*) is defined by:(66)

Hence, the probability that the *j^th ^*gene is regulated sums up to one: . Here, the score indicates that how likely a gene is regulating another one depends not only on the strength of interactions, but also on its amount.

Eventually, if *c_ij _*≥ *τ *the edge is introduced, otherwise it is omitted.

We test the asymmetric weighting on the matrix *W *inferred from the **symbolic dynamics measures**.

### ROC analysis

In order to rank the performance of the different similarity measures and scoring schemes, we evaluate to which extent each of them accurately reconstructs the underlying network of regulatory interactions. To this end, we use the receiver operating characteristic (*ROC*) analysis [60], as it provides indices to value the reconstruction efficiency among all the measures and scoring schemes under study. The *ROC *analysis is a tool for visualizing, organizing, and selecting classifiers based on their performance in terms of a cost/benefit analysis. For this purpose the *ROC *space is defined by the false positive rate, *fpr*, and the true positive rate, *tpr*, which depict the relative trade-offs between true positives *tp *(benefits) and false positives *fp *(costs). An overview on important quantities in *ROC *analysis is given in Table 3.

**Table 3:** ROC

true positives	correctly identified true edges	*tp*
false positives	spurious edges	*fp*
true negatives	correctly identified zero edges	*tn*
false negatives	unrecognized true edges	*fn*
positives	all true edges	*p *= *tp *+ *fn*
negatives	all zero edges	*n *= *tn *+ *fp*
false positive rate	part of negatives set positive	*fpr *= *fp/n*
true positive rate	part of positives set positive	*tpr *= *tp/p*
false negative rate	part of positives set negative	*fnr *= *fn/p*
true negative rate	part of negatives set negative	*tnr *= *tn/n*
recall (sensitivity)	true positive rate	*tpr*
specificity	true negative rate	*tnr*
precision	positive predictive value	*tp*/(*tp *+ *fp*)

Summary of important quantities in *ROC *analysis.

While discrete classifiers lead to just a single point in the *ROC *space, classifiers such as the similarity measures studied in this work produce probability values of how likely an instance belongs to a certain class. Here, the classification depends on a predefined threshold. The *ROC *curve is then produced by continuously tuning this threshold, which on the other hand can be suggestive on the performance of the measures. However, a well-defined rating is not always possible "by eye". Therefore, different summary statistics are common, for example the area under the *ROC *curve (*AUC*(*ROC*)) or the *YOUDEN *index (*YOUDEN *= max(*tpr *- *fpr*)) [61]. Another standard evaluation plot in the field of the *ROC *analysis is the precision/recall graph (*PvsR*), which is based on the comparison between the true edges and the inferred ones. Hence, it highlights the precision of the reconstruction, and does not suffer from the typically large number of false positives in a gene regulatory network reconstruction. We thus give the summary statistic using the area under the precision-recall curve (*AUC*(*PvsR*)) as well as the *ROC *curve. An efficient implementation of the *ROC *analysis is provided by the *R*-package "*ROCR*" [62].

All *ROC *curves are evaluated with respect to the underlying *GRN*, which is a directed graph. As several of the scoring schemes/measures do not distinguish whether the regulation is directed from gene *i *to gene *j *or vise versa, some of the false positives will follow from the missing information on the directionality. However, since the network under study is a sparse one, this additional false positives barely carry a weight.

## Competing interests

The authors declare that they have no competing interests.

## Authors' contributions

All authors participated in the selection of the measures and algorithms included in this investigation. SD and ZN have chosen the test data set (parameters). SD implemented and evaluated the measures and scoring schemes. All authors participated in the comparison, interpretation and discussion of the results. SD and AK drafted the manuscript, ZN participated in structuring and formulating of the draft. ZN and JK provided feedback. All authors wrote, read and approved the final version of the manuscript.

## Supplementary Material

Additional file 1**Supplement Figures**. Figure 1: Performance of the identity scoring scheme using different measures operating on vectors, in terms of the *ROC *curves, where the false positive rate (*fpr*) vs. the true positive rate (*tpr*) is plotted. The results shown here are obtained from the Euclidean distance (*μ_EC_*), the *Ls *norm (*μ_L_*) and the Manhattan distance (*μ_MA_*), as well as from the dynamic time warping (*μ_W_*) with the step pattern symmetric1, symmetric2 and asymmetric. Figure 2: *ROC *curves obtained for the *ID *scoring scheme using the simple, conditional and partial Pearson correlation (*μ_P_*, , ), where the diagonal of the cross-correlation matrix is set to 0, when a significance test (by reshuffling of the time series) is applied. Figure 3: Evaluation of the *ID *scoring scheme using information-theoretic measures: simple, conditional and residual mutual information (*μ_I_*,  and ) when a significance test by reshuffling is applied. Figure 4: *ROC *curves for the mutual coarse-grained information rate (, the conditional coarse-grained information rate ( (*similarity*)), and the *CCIR *represented as a distance ( (*distance*)), in frames of the identity scoring scheme. Figure 5: (a) The *ROC *curves, obtained for the simple, conditional and partial Granger causality index (*μ_G_*, , ) using the identity scoring scheme are shown. (b) The panel illustrates the associated results under consideration of significance (simple significance test by reshuffling of the time series). Figure 6: *ROC *curves obtained for the Spearman correlation coefficient *μS *using the *CLR*, *MRNET *and the *ARACNE *scoring scheme. Figure 7: Reconstruction from noisy data (noise level 0.3). *ROC *curves of (a) the Granger and partial Granger causality (*μ_G_*, ), the mutual and conditional coarse-grained information rates (, ), and the conditional mutual information (), norm, Euclidean as well as (b) the distance measures: *L^s ^*norm, Euclidean distance, Manhattan distance and dynamic time warping with the step pattern symmetric1, symmetric2 and asymmetric. Figure 8: The role of interpolation and sampling: simulated expression time series of 100 equally sampled data points (black line), the effect of (spline) interpolation (including the following data points of the original series: 1*|*2*|*3*|*6*|*9*|*15*|*25*|*39*|*63*|*99., green line). Figure 9: Artefacts introduced in the reconstruction procedure (measure: *μ_I_*, scoring scheme: *ID*) by interpolation of short, coarsely sampled time series. The left panel shows the corresponding *ROC *curves in the noise-free case for 10 points equally sampled in time, whereas the right panel presents the same results for 10 points, unequally sampled. The unequal sampling in time is the same as in Figure 8. Figure 10: *ROC *curves for selected measures and algorithms obtained in the noise-free case, using unequally sampled data without interpolation. The sampling is the same as in the previous two figures, including the following data points of a simulated series of 100 points: 1*|*2*|*3*|*6*|*9*|*15*|*25*|*39*|*63*|*99. Figure 11: *ROC *curves obtained from the reconstruction of an *E. coli *network of 100 genes, a *S.cerevisiae *network of 100 gene and an *E. coli *network of 200 genes. (a)-(i) show the results using various similarity measures together with the *ID *scoring scheme: (a) Euclidean distance *μ_EC_*, (b) Manhattan distance *μ_MA_*, (c) *Ls *norm *μ_L_*, (d) Kendall's rank correlation *μ_K_*, (e) Pearson correlation *μP*, (f) conditional Pearson correlation , (g) mutual information of symbol vectors , (h) mean of symbol sequence similarity and the mutual information of symbol vectors , and (i) conditional mutual information . Moreover, the results using Kendall's rank correlation *μ_K _*together with (j) *MRNET*, (k) *CLR*, and (l) *ARACNE *scoring scheme are shown. Figure 12: Summary statistics for the top-ranked measures/scoring schemes for increasing noise intensities (noise level 0.5). Similar approaches are grouped together. The first group in cyan refers to the different measures applied together with the *ID *scoring scheme. The green stands for the *CLR *scoring scheme, the orange for the *MRNET*, yellow refers to the *ARACNE*, magenta to the *AWE *and violet stands for the *TS*. Furthermore, blue groups together all measures applied with a combination of scoring schemes. Figure 13: Summary statistics ((a), (c) and (e) area under the *ROC *curve, as well as (b), (d) and (f) *Y OUDEN *index) for the top-ranked measures/scoring schemes as a function of the noise intensity for varying lengths of the time series. The results in (a) and (b) are obtained from 8 time points, those in (c) and (d) from 10 time points, and those in (e) and (f) from 20 time points. Figure 14: (a) Illustration of the network and its degree distribution for 100 genes in *E. coli*. Here and in the following figures *p*(*k*) is the frequency of nodes with total degree *k*, *p_in*(*k*) is the frequency of nodes with an in-degree *k*, and *p out*(*k*) is the frequency of nodes with an out-degree *k*. Futhermore, the network and its degree distribution for (b) 100 genes in *S.cerevisiae*, and (c) 200 genes in *E. coli *arClick here for file
