# Supplementary material for: Unraveling gene regulatory networks from time-resolved gene expression data -- a measures comparison study
Source: BMC Bioinformatics. 2011 Jul 19;12:292. doi: 10.1186/1471-2105-12-292 (PMC3161045; doi:10.1186/1471-2105-12-292)
Supplement: Additional file 1 — Supplement Figures. Figure 1: Performance of the identity scoring scheme using different measures operating on vectors, in terms of the ROC curves, where the false positive rate (fpr) vs. the true positive rate (tpr) is plotted. The results shown here are obtained from the Euclidean distance (μEC), the Ls norm (μL) and the Manhattan distance (μMA), as well as from the dynamic time warping (μW) with the step pattern symmetric1, symmetric2 and asymmetric. Figure 2: ROC curves obtained for the ID scoring scheme using the simple, conditional and partial Pearson correlation (μP, , ), where the diagonal of the cross-correlation matrix is set to 0, when a significance test (by reshuffling of the time series) is applied. Figure 3: Evaluation of the ID scoring scheme using information-theoretic measures: simple, conditional and residual mutual information (μI, and ) when a significance test by reshuffling is applied. Figure 4: ROC curves for the mutual coarse-grained information rate (, the conditional coarse-grained information rate ( (similarity)), and the CCIR represented as a distance ( (distance)), in frames of the identity scoring scheme. Figure 5: (a) The ROC curves, obtained for the simple, conditional and partial Granger causality index (μG, , ) using the identity scoring scheme are shown. (b) The panel illustrates the associated results under consideration of significance (simple significance test by reshuffling of the time series). Figure 6: ROC curves obtained for the Spearman correlation coefficient μS using the CLR, MRNET and the ARACNE scoring scheme. Figure 7: Reconstruction from noisy data (noise level 0.3). ROC curves of (a) the Granger and partial Granger causality (μG, ), the mutual and conditional coarse-grained information rates (, ), and the conditional mutual information (), norm, Euclidean as well as (b) the distance measures: Ls norm, Euclidean distance, Manhattan distance and dynamic time warping with the step pattern symmetric1, symmetric2 and asy [file 1471-2105-12-292-S1.PDF]

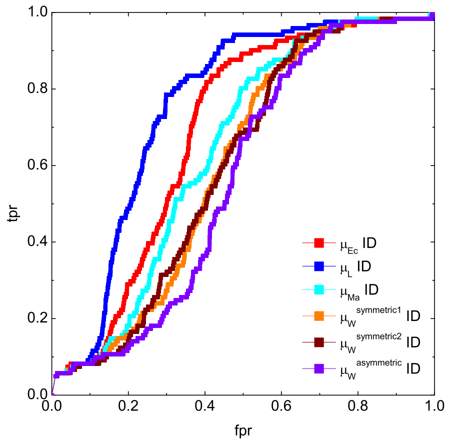

Figure 1: Performance of the identity scoring scheme using different measures operating on vectors, in terms of the *ROC* curves, where the false positive rate (fpr) vs. the true positive rate (tpr) is plotted. The results shown here are obtained from the Euclidean distance (μ_{Ec}), the L^s norm (μ_L) and the Manhattan distance (μ_{Ma}), as well as from the dynamic time warping (μ_W) with the step pattern symmetric1, symmetric2 and asymmetric.

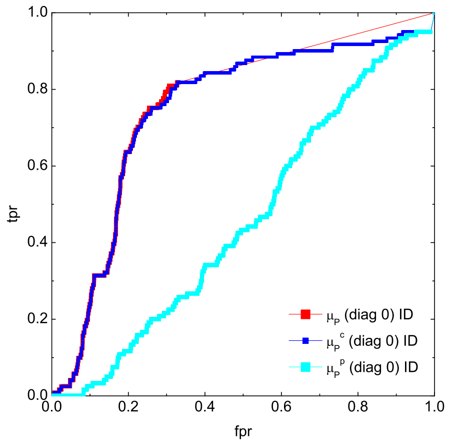

Figure 2: *ROC* curves obtained for the *ID* scoring scheme using the simple, conditional and partial Pearson correlation (μ_P, μ_P^c, μ_P^p), where the diagonal of the cross-correlation matrix is set to 0, when a significance test (by reshuffling of the time series) is applied.

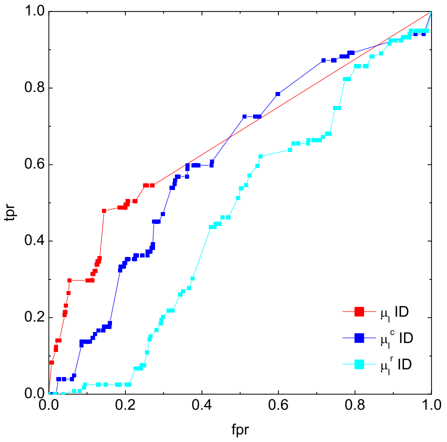

Figure 3: Evaluation of the ID scoring scheme using information-theoretic measures: simple, conditional and residual mutual information (μ_I, μ_I^c and μ_I^r) when a significance test by reshuffling is applied.

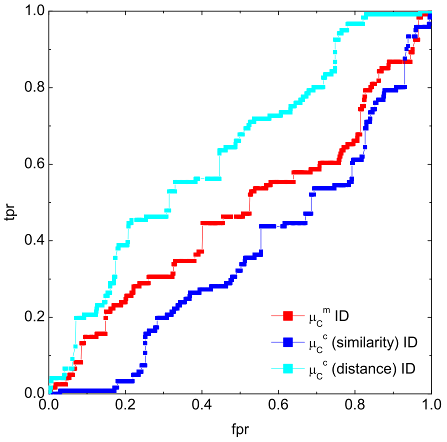

Figure 4: ROC curves for the mutual coarse-grained information rate (μ_C^m), the conditional coarse-grained information rate (μ_C^c $(similarity)$), and the $CCIR$ represented as a distance (μ_C^c $(distance)$), in frames of the identity scoring scheme.

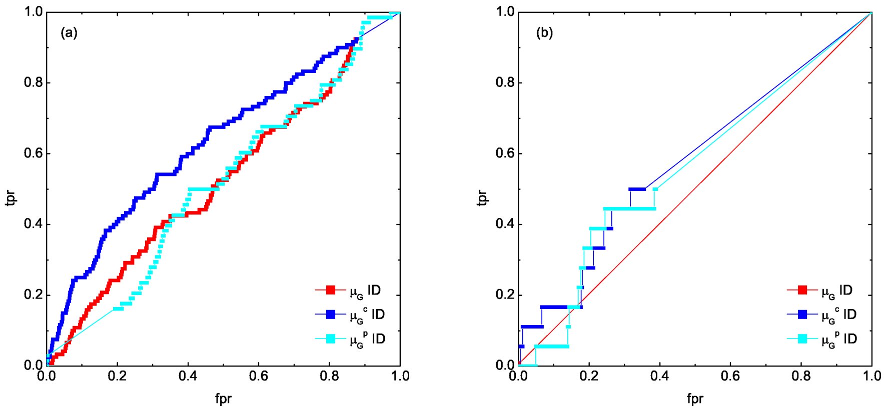

Figure 5: (a) The ROC curves, obtained for the simple, conditional and partial Granger causality index (μ_G, μ_G^c, μ_G^p) using the identity scoring scheme are shown. (b) The panel illustrates the associated results under consideration of significance (simple significance test by reshuffling of the time series).

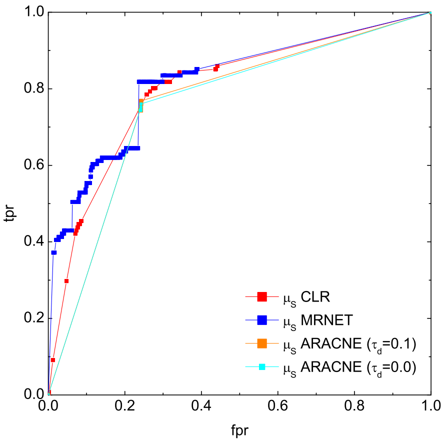

Figure 6: ROC curves obtained for the Spearman correlation coefficient μ_S using the CLR, $MRNET$ and the $ARACNE$ scoring scheme.

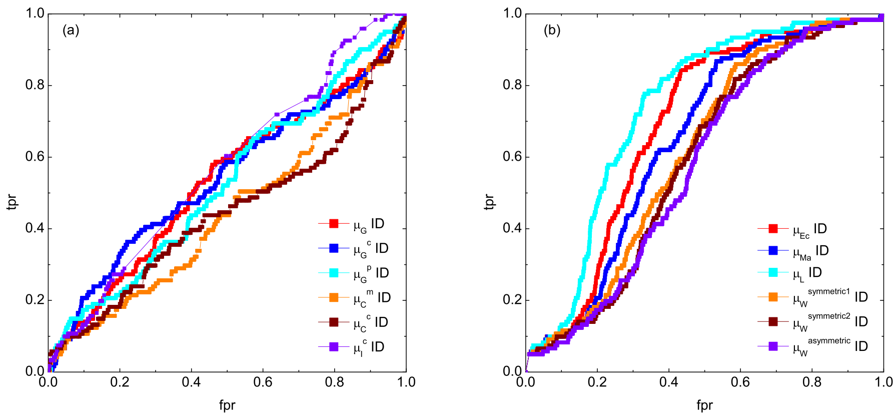

Figure 7: Reconstruction from noisy data (noise level 0.3). *ROC* curves of (a) the Granger and partial Granger causality (μ_G, μ_G^p), the mutual and conditional coarse-grained information rates (μ_C^m, μ_C^c), and the conditional mutual information (μ_I^c), as well as (b) the distance measures: L^s norm, Euclidean distance, Manhattan distance and dynamic time warping with the step pattern symmetric1, symmetric2 and asymmetric.

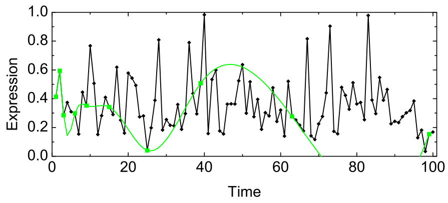

Figure 8: The role of interpolation and sampling: simulated expression time series of 100 equally sampled data points (black line), the effect of (spline) interpolation (including the following data points of the original series: 1|2|3|6|9|15|25|39|63|99, green line).

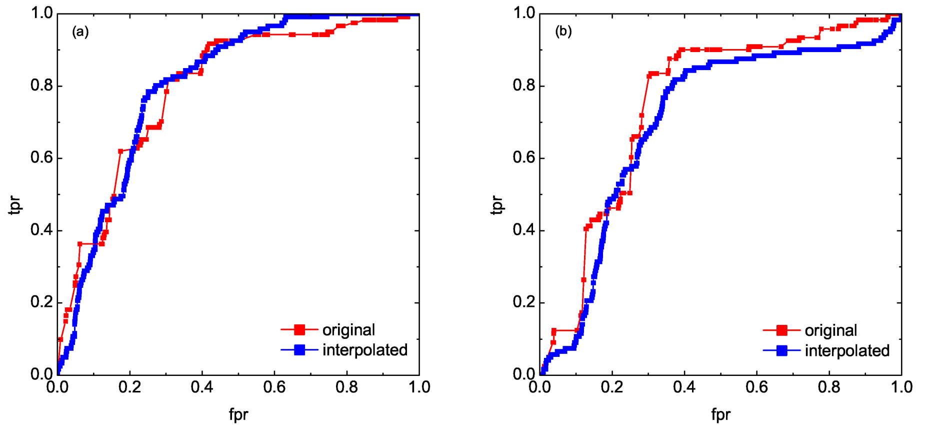

Figure 9: Artefacts introduced in the reconstruction procedure (measure: μ_I, scoring scheme: ID) by interpolation of short, coarsely sampled time series. The left panel shows the corresponding ROC curves in the noise-free case for 10 points equally sampled in time, whereas the right panel presents the same results for 10 points, unequally sampled. The unequal sampling in time is the same as in Fig. 8.

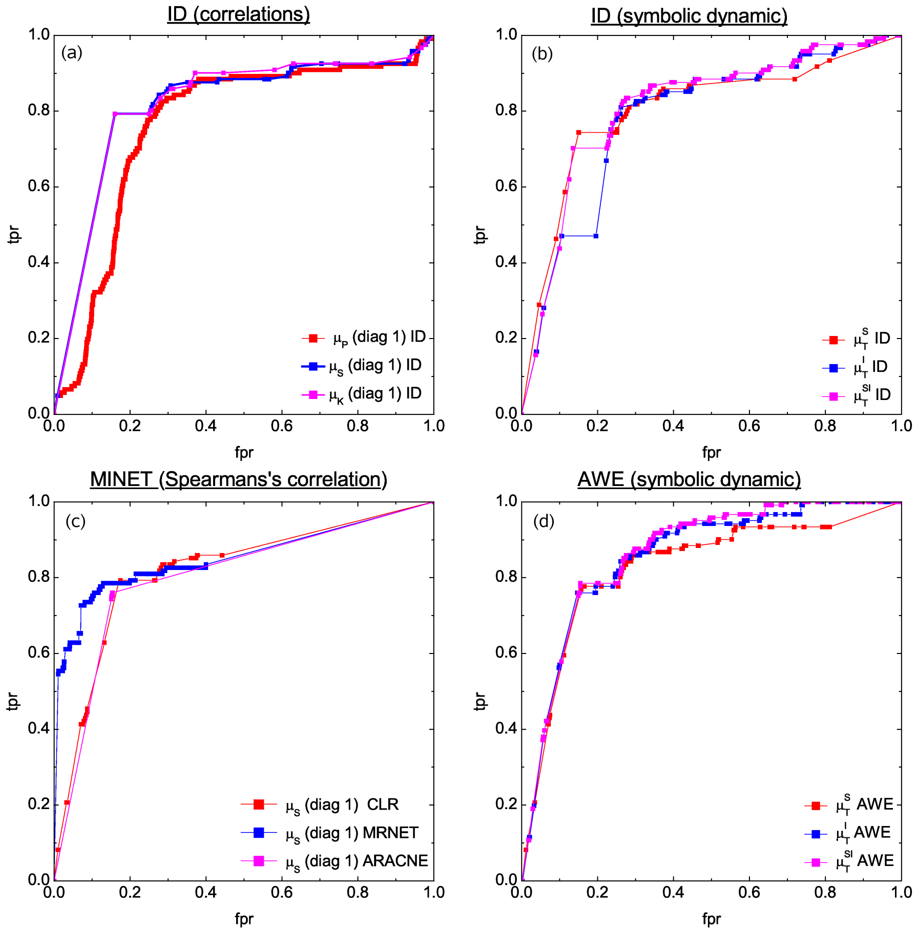

Figure 10: *ROC* curves for selected measures and algorithms obtained in the noise-free case, using unequally sampled data without interpolation. The sampling is the same as in the previous two figures, including the following data points of a simulated series of 100 points: 1|2|3|6|9|15|25|39|63|99.

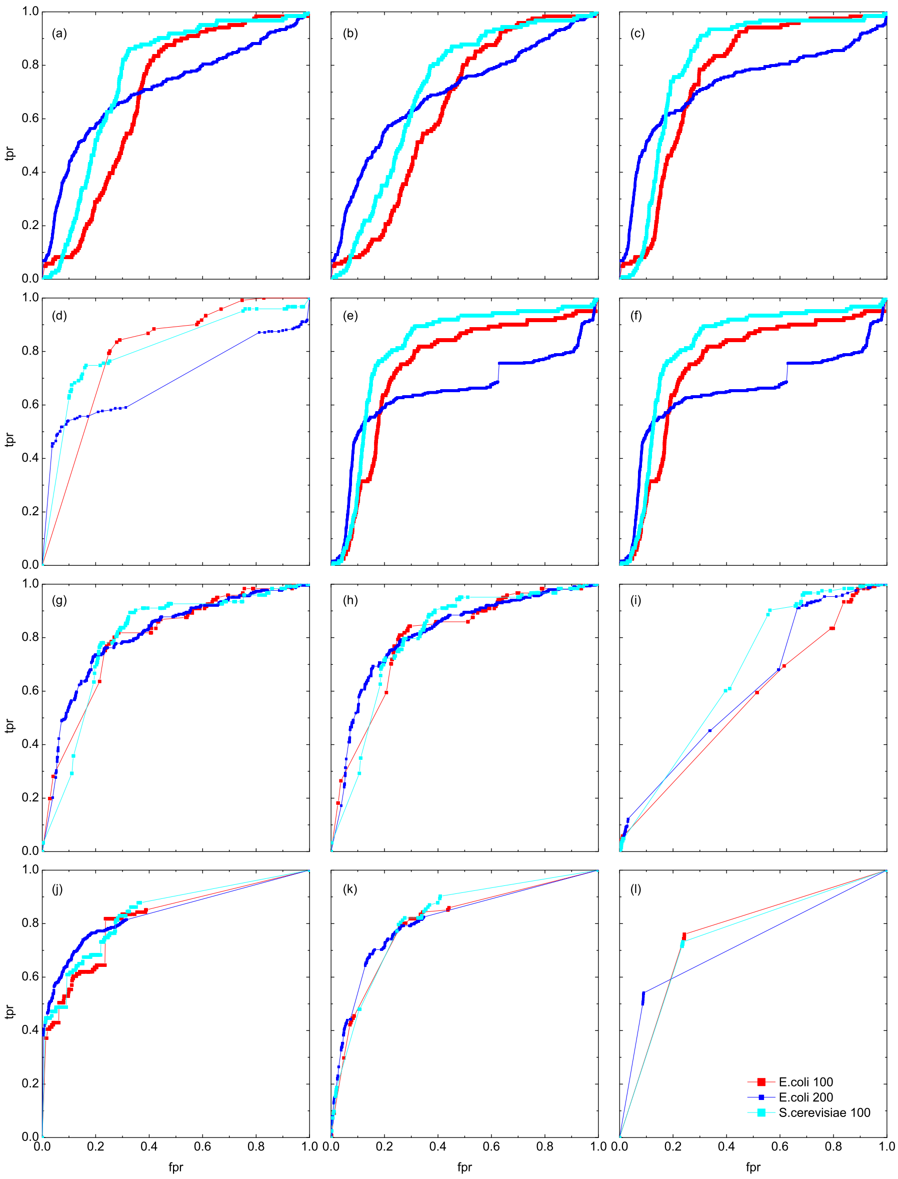

Figure 11: *ROC* curves obtained from the recontruction of an *E.coli* network of 100 genes, a *S.cerevisiae* network of 100 gene and an *E.coli* network of 200 genes. (a)-(i) show the results using various similarity measures together with the *ID* scoring scheme: (a) Euclidean distance μ_{EC}, (b) Manhattan distance μ_{MA}, (c) L^s norm μ_L, (d) Kendall's rank correlation μ_K, (e) Pearson correlation μ_P, (f) conditional Pearson correlation μ_P^c, (g) mutual information of symbol vectors μ_T^I, (h) mean of symbol sequence similarity and the mutual information of symbol vectors $\mu_T^S I$, and (i) conditional mutual information μ_I^c. Moreover, the results using Kendall's rank correlation μ_K together with (j) $MRNET$, (k) CLR, and (l) $ARACNE$ scoring scheme are shown.

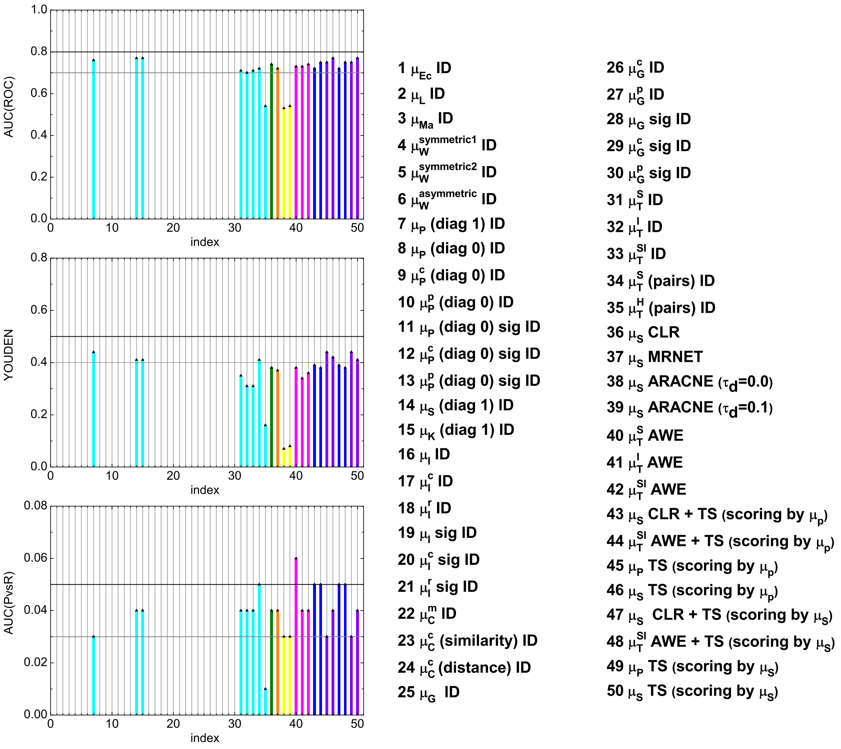

Figure 12: Summary statistics for the top-ranked measures / scoring schemes for increasing noise intensities (noise level 0.5). Similar approaches are grouped together. The first group in cyan refers to the different measures applied together with the ID scoring scheme. The green stands for the CLR scoring scheme, the orange for the $MRNET$, yellow refers to the $ARACNE$, magenta to the AWE and violet stands for the TS. Furthermore, blue groups together all measures applied with a combination of scoring schemes.

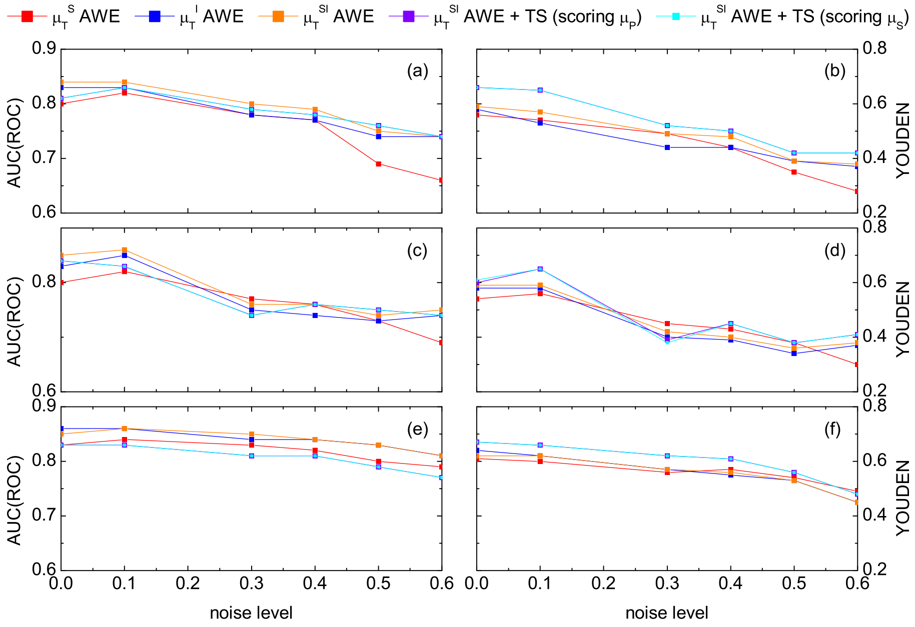

Figure 13: Summary statistics ((a), (c) and (e) area under the ROC curve, as well as (b), (d) and (f) $YOUDEN$ index) for the top-ranked measures / scoring schemes as a function of the noise intensity for varying lengths of the time series. The results in (a) and (b) are obtained from 8 time points, those in (c) and (d) from 10 time points, and those in (e) and (f) from 20 time points.

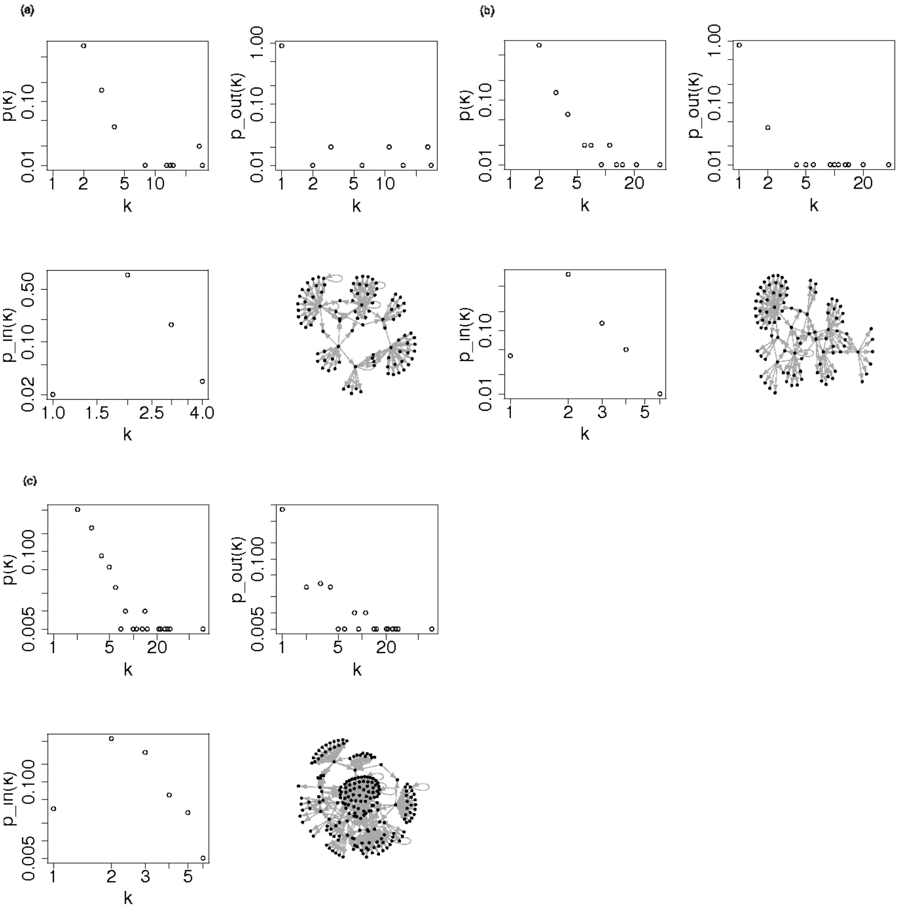

Figure 14: (a) Illustration of the network and its degree distribution for 100 genes in *E.coli*. Here and in the following figures $p(k)$ is the frequency of nodes with total degree k, $p\_in(k)$ is the frequency of nodes with an in-degree k, and $p\_out(k)$ is the frequency of nodes with an out-degree k. Futhermore, the network and its degree distribution for (b) 100 genes in *S.cerevisiae*, and (c) 200 genes in *E.coli* are shown.
